# Supplementary material for: The allotetraploid horseradish genome provides insights into subgenome diversification and formation of critical traits
Source: Nat Commun. 2023 Jul 25;14:4102. doi: 10.1038/s41467-023-39800-y (PMC10368706; doi:10.1038/s41467-023-39800-y)
Supplement: Supplementary file 1 — Supplementary Information [file 41467_2023_39800_MOESM1_ESM.pdf]

The allotetraploid horseradish genome provides insights into subgenome diversification and formation of critical traits

Shen *et al.*

Supplementary Note 1. Genome assembly using Oxford Nanopore Technology (ONT) and Hi-C data.

We conducted the genome assembly using the comprehensive and customized pipeline. First, the ONT long reads were assembled into contigs using NextDenovo software (v2.0, https://github.com/Nextomics/NextDenovo) with default parameters. The single-base accuracy was improved using Illumina reads and Pilon (v1.24) software[1]. Based on the genome survey using Illumina reads, the estimated genome size was approximately 636 million (M)bp. Initially, we assembled the long reads into 292 contigs with an N50 of 7.95 Mbp, which totaled 611 Mbp and accounted for 96% of the estimated genome size (Supplementary Table 2). Second, we employed a scaffolding approach using HiC-seq data, taking into account the possibility of chimeric assemblies caused by homoeologous contigs from the two horseradish subgenomes (Supplementary Fig. 2). To improve assembly continuity and avoid missing sequences, we implemented the following steps. First, we performed genome annotation using the MAKER-P (v2.29+) pipeline on the polished contigs, resulting in the generation of a gene set (see the detailed description of genome annotation in the Methods part)[2]. Second, we analyzed pairwise synteny and obtained collinear segments using the JCVI (v1.2.20) software[3]. The pairwise *Ks* values of the homoeologous genes were calculated using PAML (v4.9j)[4]. Third, based on the *Ks* values, we predicted synteny blocks derived from the two different subgenomes. HiC-seq reads were aligned to contigs using Juicer (v1.6)[5]. Paired read links between the predicted homoeologous contigs from the two subgenomes were removed. Finally, we used the 3D *de novo* assembly (3D-DNA)[5] pipeline (v201008) for scaffolding.

We partitioned and phased the subgenomes based on repetitive *k-mer*s and orthologous genes using SubPhaser (v1.2)[6]. To avoid the effects of large-scale genomic deletions on clustering, we used only chromosomal regions within the syntenic region for analysis. We ran the SubPhaser with different parameters ('-k 13 -q 100 -f 2', -k 15 -q 100 -f 2', '-k 15 -q 200 -f 2', '-k 13 -q 200 -f 2'). In addition, the orthologous genes were also examined. We adopted the branch length of the phylogenetic tree constructed based on the orthologous genes. The *Arabidopsis thaliana* genome was used to determine the synteny block and orthologous gene pairs with each scaffold using JCVI software[3]. Orthologous gene pairs were subjected to multiple sequence alignment using MAFFT (v7.490) software with default parameters[7] and concatenated within each scaffold. Phylogenetic trees were constructed based on the concatenated sequences using FASTTREE (v2.1.11) with default parameters[8].

Multiple methods were used to assess the quality of the genome assembly. The read re-mapping ratio was assessed using Illumina reads and ONT long reads. Illumina short reads were aligned to the genome using BWA MEM (v0.7.17) software with default parameters[9], while RNA reads were aligned using Hisat2 (v2.2.1) software with default parameters[10]. Minimap2 (v2.24)[11] software with parameters "-a -x map-ont -t 30" was used to map the long reads. The integrity of the genetic region was assessed using BUSCO (v4.0) software[12]. In addition, the quality of repeat sequences was assessed using the LTR Assembly Index (LAI)[13].

Karyotypes of Brassicaceae species were determined by comparative chromosome painting (CCP) by Lysak and his colleagues[14–16]. The concept of Ancestral Crucifer Karyotype (ACK; $n = 8$)

containing 22 genomic blocks (GBs, named A to X) was proposed[17]. To analyze the horseradish karyotype, we extracted the GBs from the *A. thaliana* genome and performed collinearity analysis between the genomes. We observed that nearly all ancestral GBs could be traced in the horseradish genome. However, there were also differences between the assemblies of the two subgenomes (Supplementary Fig. 6), such as losses of large genomic segments (blocks F and R). We constructed the horseradish karyotype based on the ancestral GBs and compared it with the karyotype generated by CCP[18]. We observed six ancestral chromosomes (AK1, AK2, AK3, AK4, AK5, and AK7) in both subgenomes (Supplementary Fig. 6). Two chromosomes (a/b06 and a/b08) originated through a reciprocal translocation involving ancestral chromosomes AK6 and AK8, in accordance with the previously published cytogenomic map[18] (Supplementary Fig. 23). The partial losses of GB F on chromosome a03 and GB R on chromosome b08 have not been detected in the comparative cytogenomic map (Supplementary Fig. 6)[18]. To elucidate this discrepancy, we performed CCP validation of the two genomic blocks. As two genomic copies of both GBs were identified by CCP (Supplementary Fig. 7), we concluded that the purported deletions were due to an incomplete genome assembly (Supplementary Fig. 7).

Supplementary Note 2. Genome assembly using PacBio HiFi and Hi-C data.

To further improve the genome assembly, we performed genome sequencing and generated PacBio HiFi long reads[19]. The tender leaves of horseradish were harvested and used for DNA extraction and sequencing library construction. The SMRTbell libraries were prepared according to the standard protocol of PacBio (Pacific Biosciences) and sequenced on the PacBio Sequel II platform to generate HiFi reads[20,21]. Finally, we obtained 27.71 Gbp of consensus reads after processing (Supplementary Table 1). The contigs from HiFi reads were assembled using HiFiasm (v0.17.7) software with default parameters[22]. Ultimately, we obtained a HiFi-based genome assembly with a genome size of 612 Mbp and an N50 length of 34 Mbp (Supplementary Table 5). We aligned the contigs to the ONT-based genome assembly using minimap2[11] and generated the dot plot using the dotPlotly software (https://github.com/tpoorten/dotPlotly). The dot plot showed that the missing genomic blocks in the ONT-based genome assembly were present in the HiFi-based genome assembly (Supplementary Fig. 8), indicating improved integrity and continuity of the HiFi assembly. Taking advantage of the accuracy of the long HiFi reads and the high continuity of the assembly, we directly performed scaffolding. This resulted in a genome assembly comprising 16 pseudochromosomes that anchored 97.29% of the assembled sequence, leaving only 8 gaps (Supplementary Table 6). Notably, nine of the 16 pseudochromosomes consisted of a single contig, highlighting the advantages of high-accuracy HiFi reads in resolving complex polyploid genomes (Supplementary Table 6).

Considering the improved continuity and integrity of the HiFi-assembled genome, we selected it as the genome backbone and filled the remaining gaps using the ONT-based genome with TGS-gapcloser (v1.2.1) software[23]. After closing all gaps, we obtained a gap-free reference genome consisting of 16 chromosomes with the total length of 610 Mbp. By searching for the seven-base telomere repeat sequence (CCCTAAA), we identified 31 telomeres (15 pairs plus one singleton) and constructed 15 T2T pseudomolecules (Supplementary Table 7). The quality of the genome

assembly was assessed using methods described earlier (see Supplementary Note 1).

Supplementary Note 3. Identification of intact (retro)transposons.

Due to the high abundance of LTR retrotransposons in the horseradish genome, we investigated the dynamics of LTR retrotransposons during genome evolution. First, we used the LTR-retriever software (v2.9.0)[24] to integrate the outputs from LTRharvest (v2.9.0)[25] and LTR_FINDER (v1.07)[26] using default parameters. A total of 16,977 intact (retro)transposons were identified in the horseradish genome, consisting of 5,695 DNA transposons and 11,282 LTR-RTs (Supplementary Table 16). The distribution of intact (retro)transposons was similar in both subgenomes (Supplementary Data 2). Analysis of the insertion timing of LTR-RTs across the genome showed that recent bursts of different LTR-RT types occurred ~0.2 million years ago (Fig. 2c). In addition, we observed variation in burst timing between different LTR-RT types (Fig. 2c), possibly due to differences in transpositional activity and/or epigenetic status of the two LTR-RT superfamilies.

Supplementary Note 4. Clustering of intact LTR-RTs and characterization of LTR-RT families.

To further characterize the LTR-RTs family and classify the clades among different LTR-RT families, we developed an in-house pipeline volcano (https://github.com/maypoleflyn/valcano). In brief, accurate identification of LTR-RTs was conducted using LTR-retriever[24]. The LTR-RTs were classified based on the identity of LTR sequences using CD-HIT-EST (v4.8.1)[27] with parameters '-c 0.8 -aL 0.8 -T 0 -M 0 -n 5 -d 200'. RepeatMasker (v4.1.2) software was used with the clustered LTR-RT family sequences as a library to determine the copy number and coverage of each family in the genome. To determine the phylogenetic relationship of the identified LTR-RTs, the reverse transcription domains of different types of LTR-RTs were used as queries to search and obtain reverse transcription domain sequences for each LTR-RT element using tBLAST[28]. The obtained amino acid sequences of reverse transcription domains were subjected to multiple alignments using MAFFT (v.7.0)[7] with default parameters. Phylogenetic trees were inferred using FASTTREE (v.2.1)[8] with default parameters and visualized using ITOL (https://itol.embl.de/). Expression of transposable elements was estimated using Telescope software (v1.0.3)[29]. RNA-Seq reads were aligned to the genome assembly using HISAT2 software[10]. The annotation GTF file was obtained from the LTR-retriever.

In our results, we classified the full-length LTR-RTs into 1,949 families, with an average of 5.8 members per family (Supplementary Data 3). Notably, the 67 families with the highest number of members accounted for more than 50% of all full-length LTR-RTs (Supplementary Data 3). To construct phylogenetic trees, we extracted a total of 3,531 Ty3/*Gypsy* and 2,842 Ty1/*Copia* retrotransposons, resulting in the identification of nine lineages consistent with similar studies in other plants[30]. The Ty1/*Copia* retrotransposons were categorized into four lineages (*Ale*, *Ivana*, *Maximus*, and *Tork*), while the Ty3/*Gypsy* retrotransposons formed five clades (*Athila*, *CRM*, *Galadriel*, *Reina*, and *Tekay*) (Fig. 2d, Supplementary Fig. 14). Among the Ty1/*Copia* retrotransposons, the most abundant were *Ale* elements (51.4%), followed by *Tork* (35.16%), *Maximus* (10.83%), and *Ivana* (2.60%). In Ty3/*Gypsy* retrotransposons, the *CRM* clade occupied approximately 54.93% of the sequences, followed by the clades *Tekay* (18.53%), *Athila* (16.51%),

Reina (6.38%), and *Galadriel* (3.65%).

We used RepeatMasker with a custom library created from clustered LTR-RT family sequences to assess the coverage of each LTR-RT family in the genome. Our analysis revealed that the repetitive nature of the genome is primarily influenced by specific LTR-RT families exhibiting exceptionally high genome coverages (Supplementary Data 3, Supplementary Fig. 15). Notably, the 150 LTR-RT families with the highest genome coverage accounted for nearly 50% of the total LTR-RT genome coverage (Supplementary Data 3). Among these families, FAM1 classified as 'unknown' LTR-RTs, had the highest coverage (~2.88%) (Supplementary Data 3). All clustered sequences of LTR-RT families can be found on github (https://github.com/maypoleflyn/HG).

Supplementary Note 5. The influence of the intact LTR-RTs on nearby genes.

We performed further studies to examine the influence of intact LTR-RTs on nearby genes. A total of approximately 1,497 genes were found in close proximity (<1000 bp) to intact *Gypsy*/*Copia* LTR-RTs (Supplementary Fig. 16a, b). To assess the expression levels near different types of LTR-RTs, we calculated the average expression value in the different tissues (root, stem, and leaf) using the deeptools software with a sliding window size of 50 bp. Our results showed that LTR-RTs, particularly *Gypsy* LTR-RTs, had a negative impact on the expression levels of nearby genes (Supplementary Fig. 16a, b). Consequently, we observed increased methylation levels in the vicinity of LTR-RTs, with *Gypsy* LTR-RTs having significantly higher methylation levels compared to the *Copia* type (Supplementary Fig. 16e, f). In addition, we quantified the expression of different retrotransposons and found a higher proportion of individuals with high expression levels among *Copia* LTR-RTs (Supplementary Fig. 16g). KEGG enrichment analysis of genes located near different types of LTR-RTs (within 1,000 bp) indicated their association with crucial metabolic pathways/BRITE hierarchies such as glutathione metabolism, starch and sucrose metabolism, biosynthesis of various plant secondary metabolites, fatty acid degradation, and lipid metabolism (Supplementary Fig. 16h, i). Based on these findings, we propose that LTR-RTs may exert a negative influence on nearby gene expression by increasing methylation levels, thereby affecting certain biological processes.

Supplementary Note 6. The influence of fragmented LTR-RTs on DNA methylation.

Fragmented LTR-RTs occupied a dominant proportion in terms of their abundance in the genome compared to intact and active LTR-RTs. Based on the observation of increased methylation levels in the vicinity of intact LTR-RTs, we extended our investigation to the methylation levels in the vicinity of fragmented LTR-RTs. Using the sequences of full-length LTR-RTs as a library, we ran RepeatMask software to identify and annotate fragmented LTR-RTs. The fragmented LTR-RTs were classified into three groups (group 1: <30%; group 2: >=30% and <=60%; group 3: >60%) based on coverage (the length of fragmented LTR-RTs/the length of corresponding intact LRT-RTs). Our observations revealed significantly increased methylation levels in different types/groups of fragmented LTR-RTs. Among flanking sequences, group 1 had the lowest methylation level, while higher methylation levels were observed in groups 2 and 3 (Supplementary Fig. 32). We then examined only the fragments with coverage greater than 50% and divided these fragments into three

classifications based on sequence identity (Classification I: >70% and <80%; Classification II >=80% and <=90%; Classification III: >90% and <100%). The methylation level of the fragmented LTR-RTs also increased, with sequences highly similar to intact LTR-RTs showing higher methylation levels (Supplementary Fig. 33). Based on these observations, it is suggested that the high methylation levels in the LTR-RTs persist even in the absence of their activity.

Supplementary Note 7. The influence of fragmented LTR-RTs on nearby genes.

To investigate the impact of fragmented LTR-RTs on genes, the occurrence of high-confidence fragmented LTR-RTs longer than 200 bp was calculated in different genic regions, including exons, introns, and upstream regions (-500 bp). The analysis revealed that fragmented LTR-RTs were present in genic regions of 9,336 genes. Specifically, they were found in the exons of 1,769 genes, in the introns of 2,383 genes, and in the upstream regions within 500 bp of 6,951 genes (Supplementary Fig. 34). These results suggest that a large number of the affected genes may be affected by alterations in gene structure or expression due to the presence of fragmented LTR-RTs. Examination of syntelogs between the two subgenomes identified a total of 3,683 genes that harbored fragmented LTR-RTs specific to the A-subgenome, and 3,676 genes that contained fragmented LTR-RTs specific to the B-subgenome (Supplementary Fig. 34). Thus, fragmented LTR-RTs play a crucial role in subgenome diversification.

Analysis of fragmented LTR-RTs in the formation of differentially methylated syntelog pairs between subgenomes revealed that approximately 24.04% (or 24.70%) of syntelog pairs with a significantly higher methylation level in the A (or B) subgenome contained fragmented LTR-RTs exclusively in the A (or B) subgenome (Supplementary Fig. 34). This suggests a possible link between the presence of fragmented LTR-RTs and the observed different methylation patterns in the subgenomes, suggesting their possible role in shaping subgenome-specific methylation profiles.

Supplementary Note 8. Analysis of centromeric regions.

Plant centromeres are usually composed of repetitive sequences, including LTR-RTs and tandem repeats[31]. In the *A. thaliana* genome, centromeres contain megabase-long islands of 178-bp tandem satellite DNA repeats (CEN180) and *Athila* retrotransposons[31–33]. The Hi-C interaction heatmap surrounding centromeric regions may have a lack signals, commonly referred to as "blank regions", due to the high density of repeats. Sequencing of repetitive regions with high GC content remains challenging using the Illumina platform[34,35]. In addition, aligning sequence reads of high repetitive content is difficult[34,35]. To predict centromeric regions in the horseradish genome, we integrated the Hi-C interaction heat map, the density distribution of satellite DNA repeats, and the density distribution of LTR-RTs. The LTR-RTs were predicted using RepeatMasker with a custom library constructed by the EDTA pipeline[36]. Tandem repeats with monomer lengths ranging between 80 and 2000 bp were identified using TRF software[37], using parameters "2 7 7 80 10 50 2000 -f -d -m -l 15". A sliding window approach with a window size of 100 kbp and a sliding step size of 100 kbp was used to calculate the density of repeats in the genome. Using the density of repeats and the Hi-C interaction heat map, we estimated the approximate location of 16 centromeric regions, ranging from 2.8 to 18.5 Mbp with an average length of 5.26 Mbp (Supplementary Figs. 10-13,

Supplementary Table 8).

To determine the repetitive elements in the centromeric regions, clustering of repeat monomers was performed for each chromosome using CD-HIT-EST software[27]. Tandem repeat monomers showing more than 80% similarity were grouped into single-sequence clusters. The most abundant tandem repeat clusters within the centromeric regions were identified as the most abundant tandem repeat monomers in the centromeric regions (Supplementary Data 4). We observed that the "blank region" on the Hi-C interaction map contained a significantly high density of tandem repeats, particularly in the centromeric regions. This observation is consistent with findings from other T2T genome assemblies, such as the kiwifruit[38] and faba bean[39] genomes. Specifically, in chromosome b04, we identified a super-long centromeric region (~18.5 Mbp) harboring megabase-long islands of four highly abundant tandem repeats (CEN194-1, CEN194-2, CEN194-3, CEN195) (Supplementary Data 4). In contrast, we did not observe similar super-long centromeric regions or such highly abundant tandem repeats in the centromere of chromosome a04, indicating significant divergence of the homoeologous centromeric regions. To further investigate the structure of the centromeric regions, we used an approach similar to that described for the human genome using StainedGlass software[40,41] and visualization of the complex tandem repeats within the centromeric regions revealed the distinctiveness of the high-order structures the two subgenomes (Supplementary Figs. 17-18). Methylation levels in the vicinity of centromeric regions were strikingly high, consistent with the presence of repetitive elements and previous studies of centromeric regions (Supplementary Fig. 19).

We scanned the five most abundant LTR-RT families in centromeric regions of all chromosome. We found that certain LTR-RT families with the highest coverage were found on different chromosomes. For instance, FAM7 (unknown classification) was found on 12 different chromosomes, while FAM13 was found on seven different chromosomes (Supplementary Data 5). LTR-RT families with the highest coverage belonged to *Gypsy*-*CRM*, *Copia*-*Ale* or unknown classification. Interestingly, we found that FAM1 (unknown classification) occupied approximately 35.58% of the centromeric region on chromosome b04, a significantly higher ratio compared with other chromosomes. In contrast, FAM16 (*Gypsy-CRM*) occupied nearly 11.47% of the centromeric region on the homoeologous chromosome a04 (Supplementary Data 5). Based on these findings, we hypothesize that the combination of the high density of CEN194/195 and the presence of FAM1 contributed to the origin of the super-long centromeric region on chromosome b04.

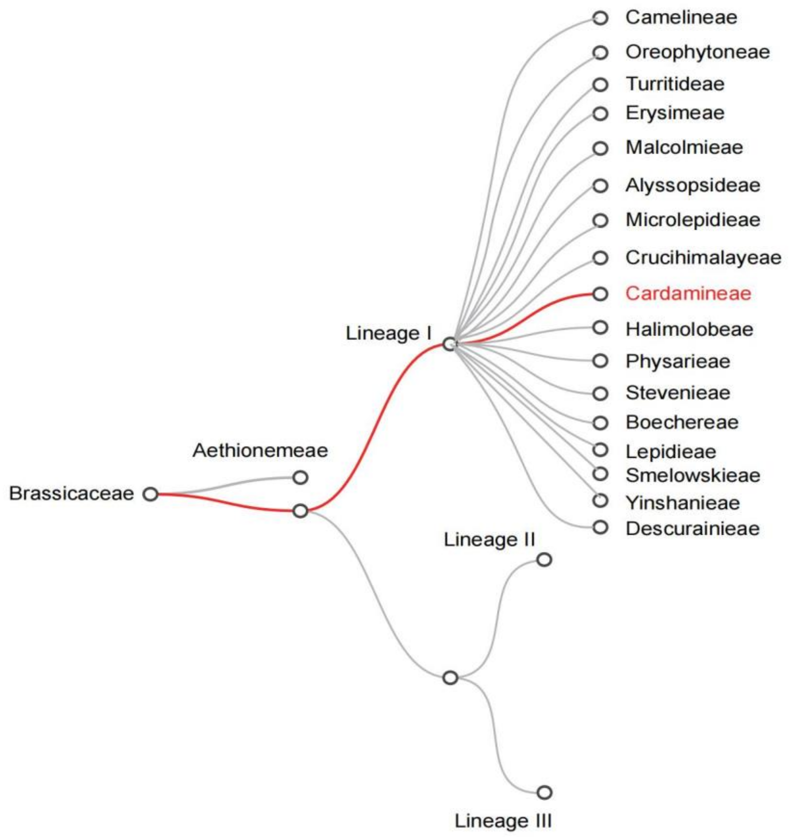

Supplementary Fig. 1. The phylogenetic position of horseradish.

The phylogenetic scheme was drawn based on the phylogenetic relationship described by Walden *et al.*[42]. The location of Cardamineae was highlighted by red lines.

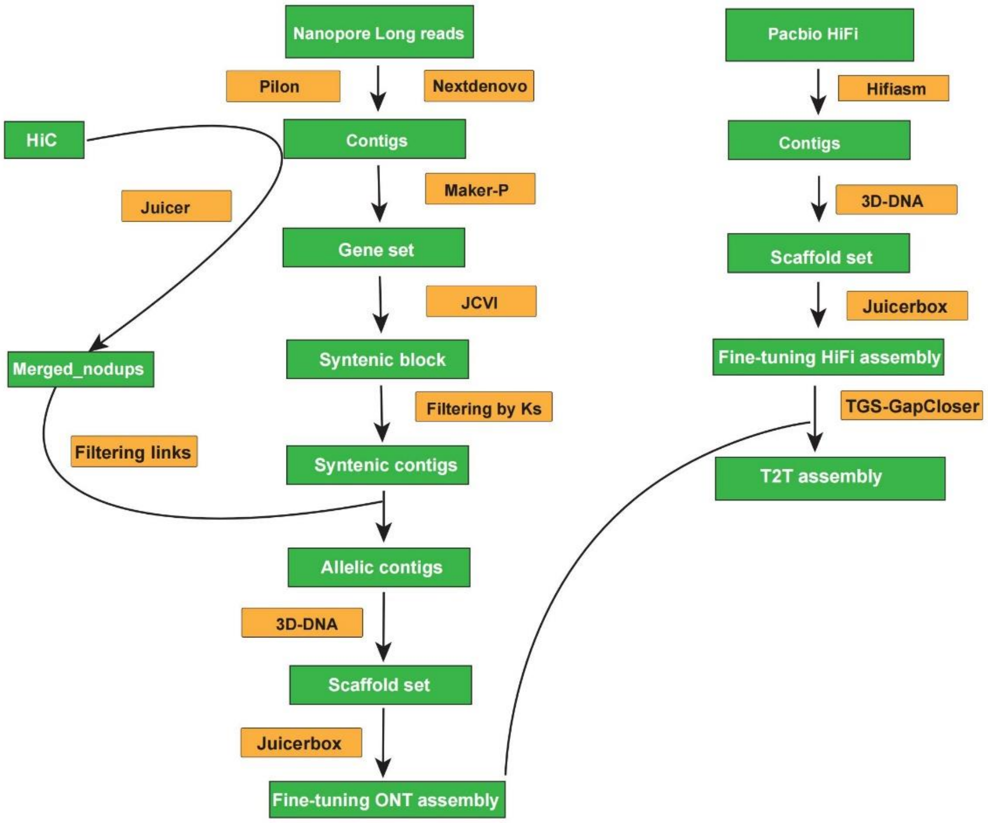

Supplementary Fig. 2. Flowchart of the customized genome assembly for the allotetraploid horseradish.

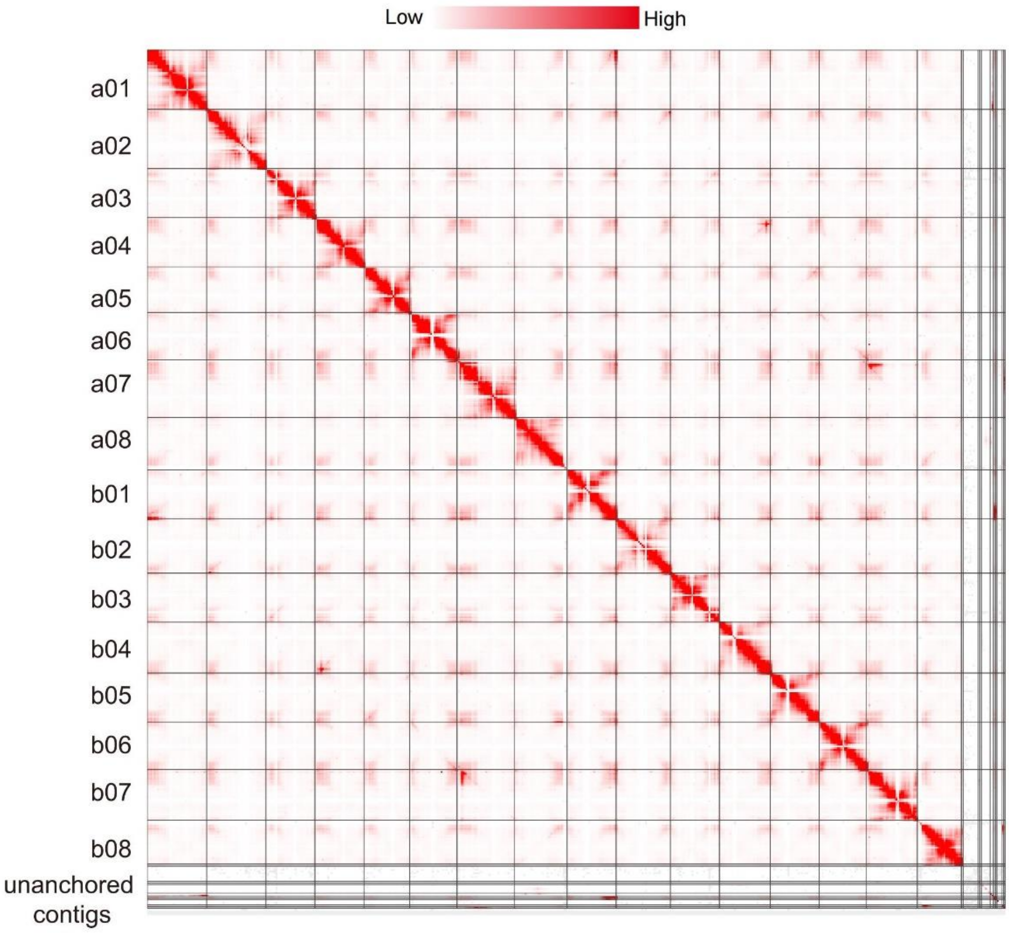

Supplementary Fig. 3. Hi–C interactions within the ONT-based genome assembly of horseradish with a 500-kb resolution.

ONT: Oxford Nanopore Technology

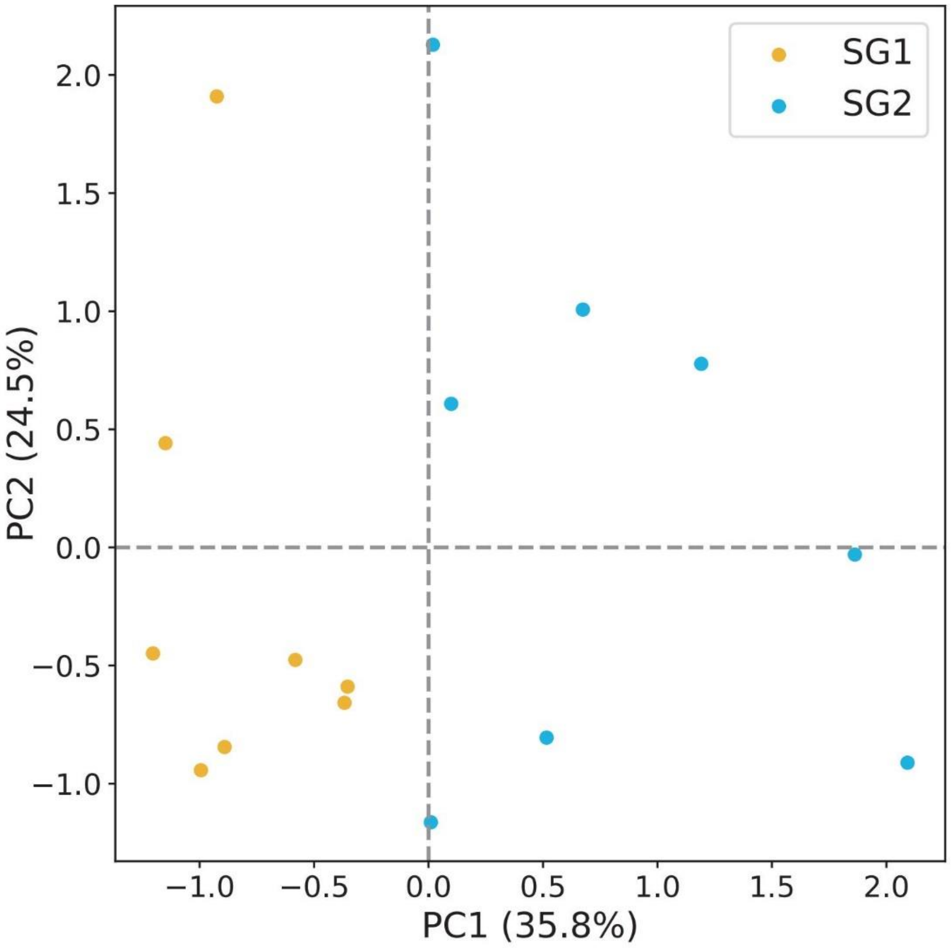

Supplementary Fig. 4. Principal component analysis (PCA) of differential *k*-mers.

Points indicate chromosomes. SG1: Asubgenome; SG2: B subgenome.

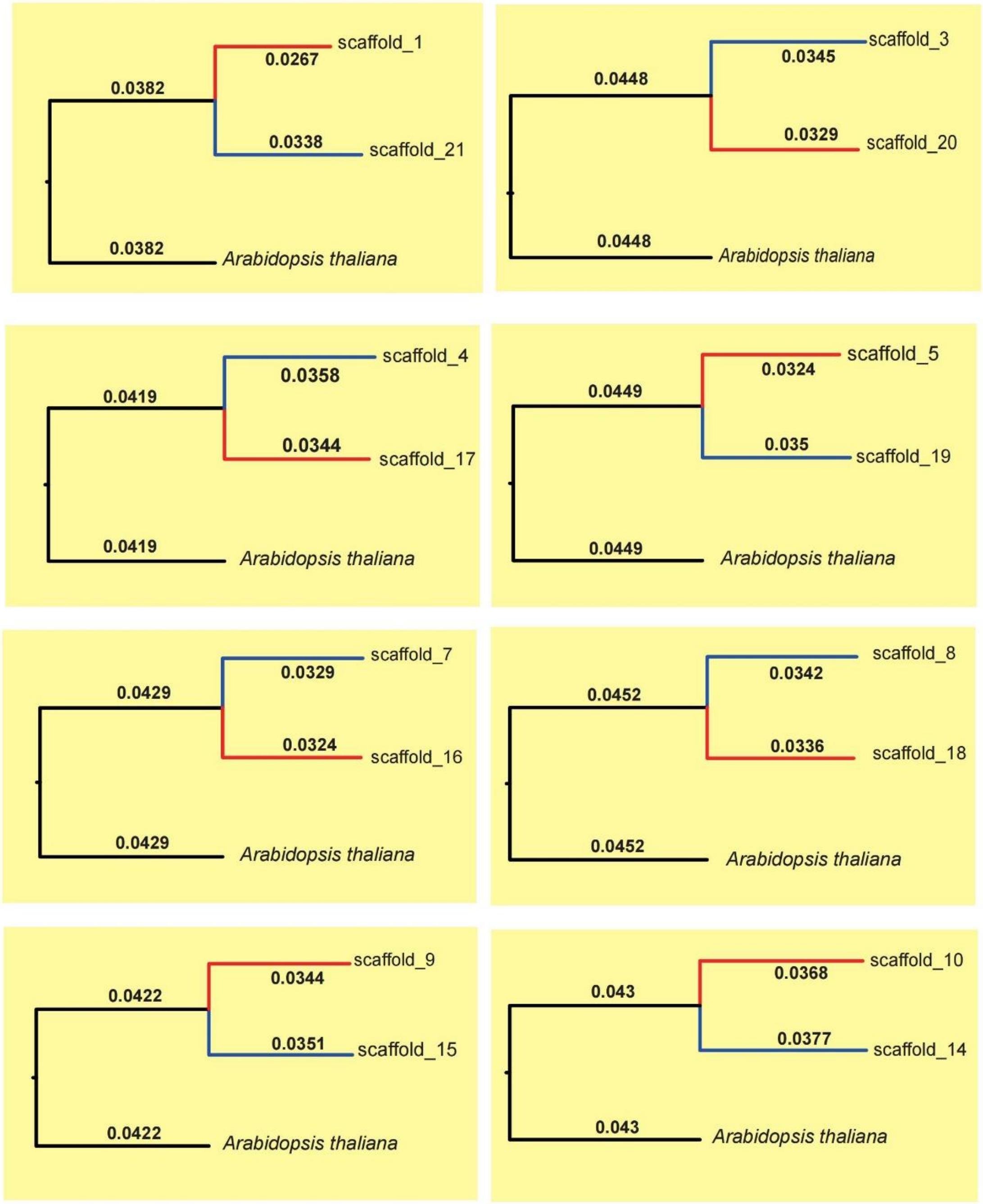

Supplementary Fig. 5. The partition and phasing of horseradish subgenomes. We used the branch length of the phylogenetic tree based on the orthologous genes to partition subgenomes. The *Arabidopsis thaliana* genome was used to identify synteny blocks and orthologous gene pairs for each scaffold. Orthologous gene pairs were subjected to multiple sequence alignment and concatenated within each scaffold. Phylogenetic trees were then built based on the concatenated sequences. red: A subgenome; blue: B subgenome.

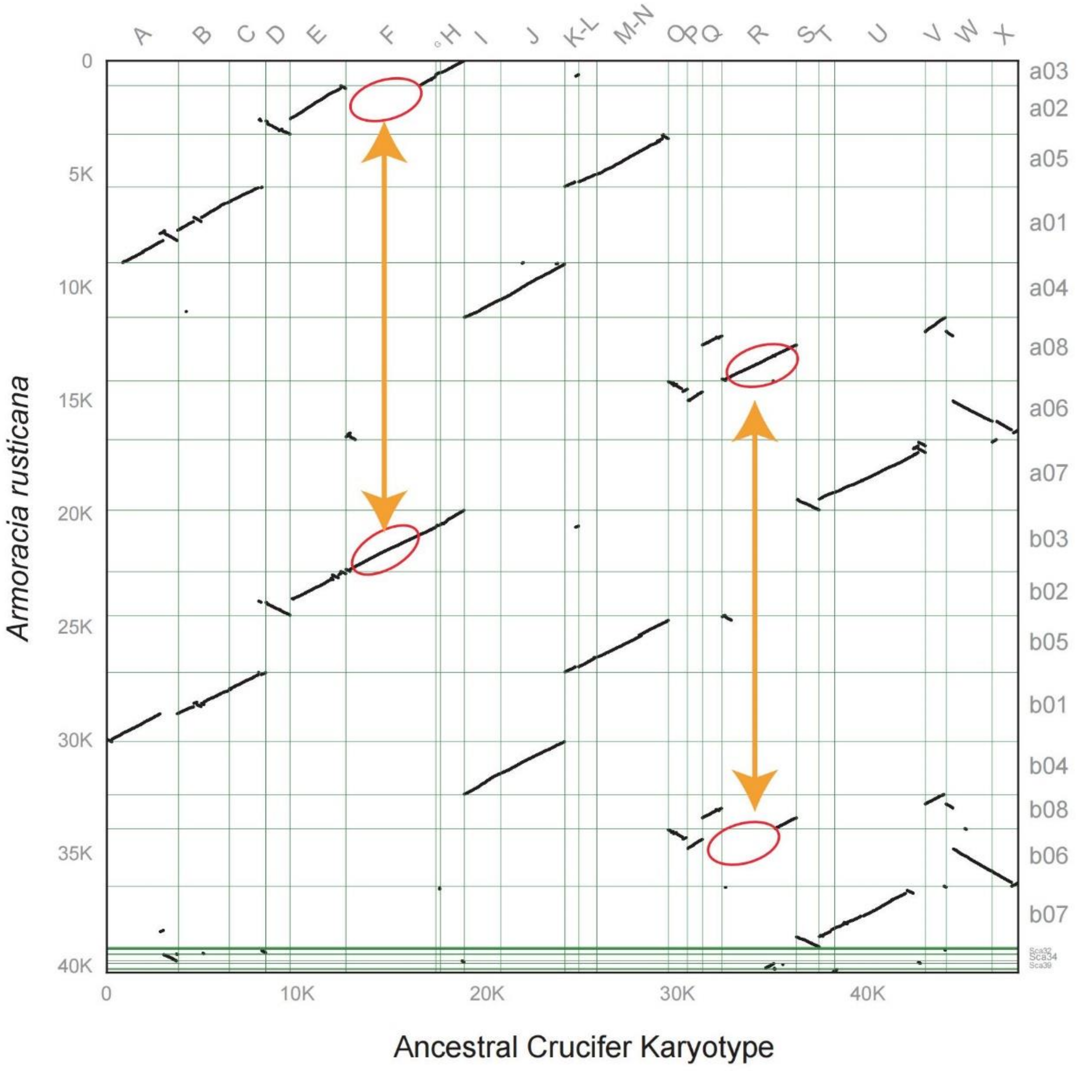

Supplementary Fig. 6. Pairwise synteny visualization of Ancestral Crucifer Karyotype and the genome assembly of *Armoracia rusticana.*

A-X: The Ancestral Crucifer Karyotype (ACK) comprising 22 genomic blocks.

The presumably lost large genomic sections are highlighted.

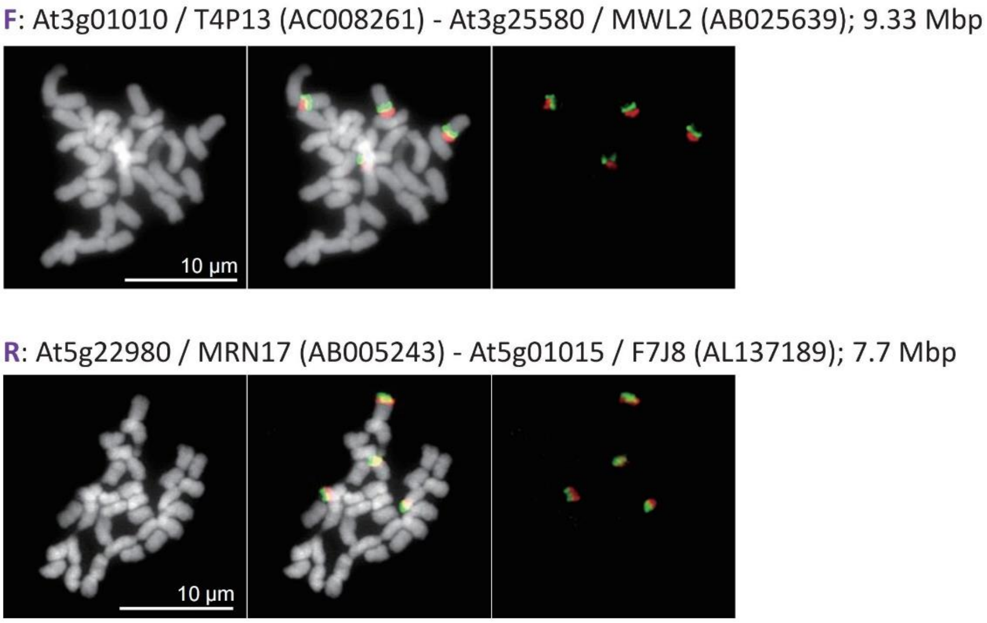

Supplementary Fig. 7. Confirmation of the presence of duplicated genomic blocks F and R by comparative chromosome painting using *Arabidopsis thaliana* BAC clones on mitotic chromosomes of *A. rusticana*.

Three times the experiment was repeated independently with similar results. scale bar, 10 µm.

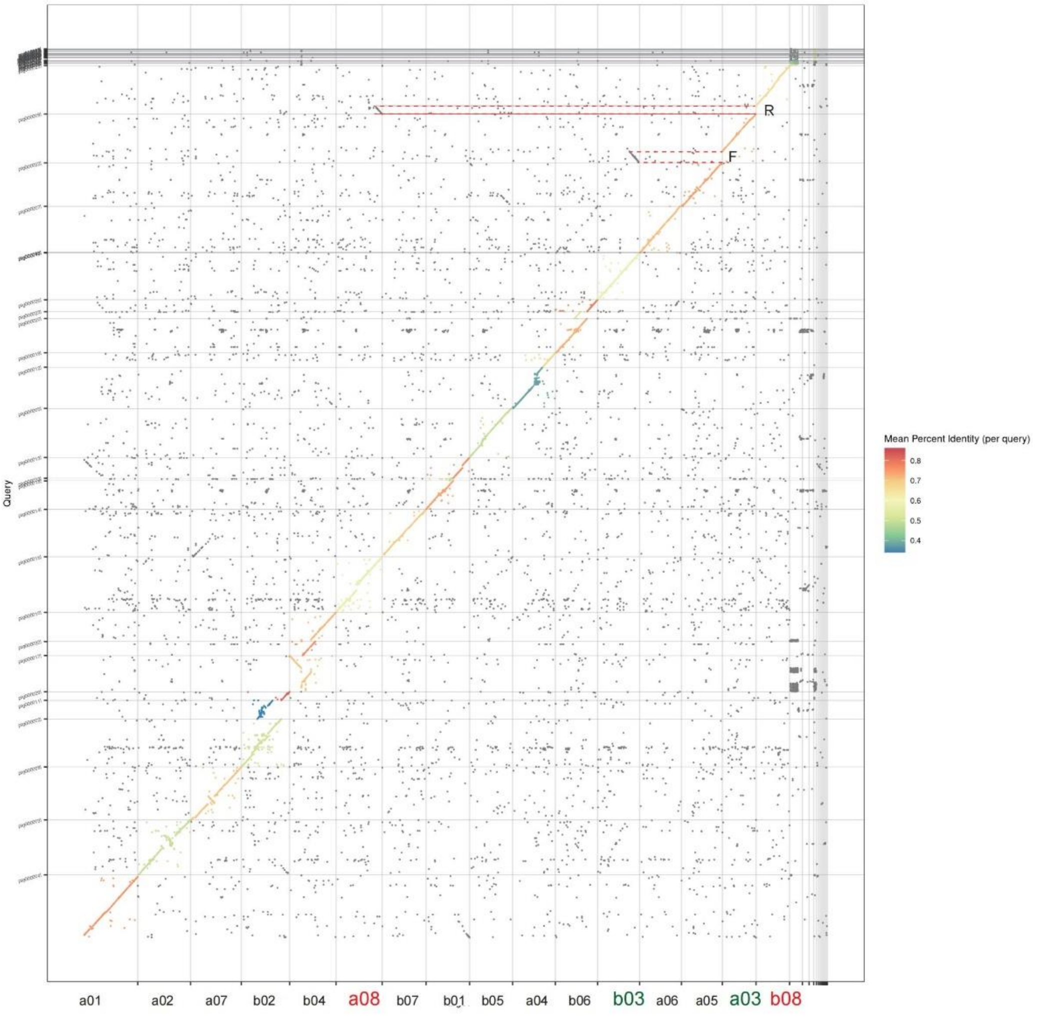

Supplementary Fig. 8. Pairwise comparison of contigs obtained using PacBio HiFi with the chromosome-level genome assembly based on ONT and HiC sequencing.

x-axis: chromosome-level genome; y-axis: PacBio HiFi contigs.

The presumably lost segments are highlighted.

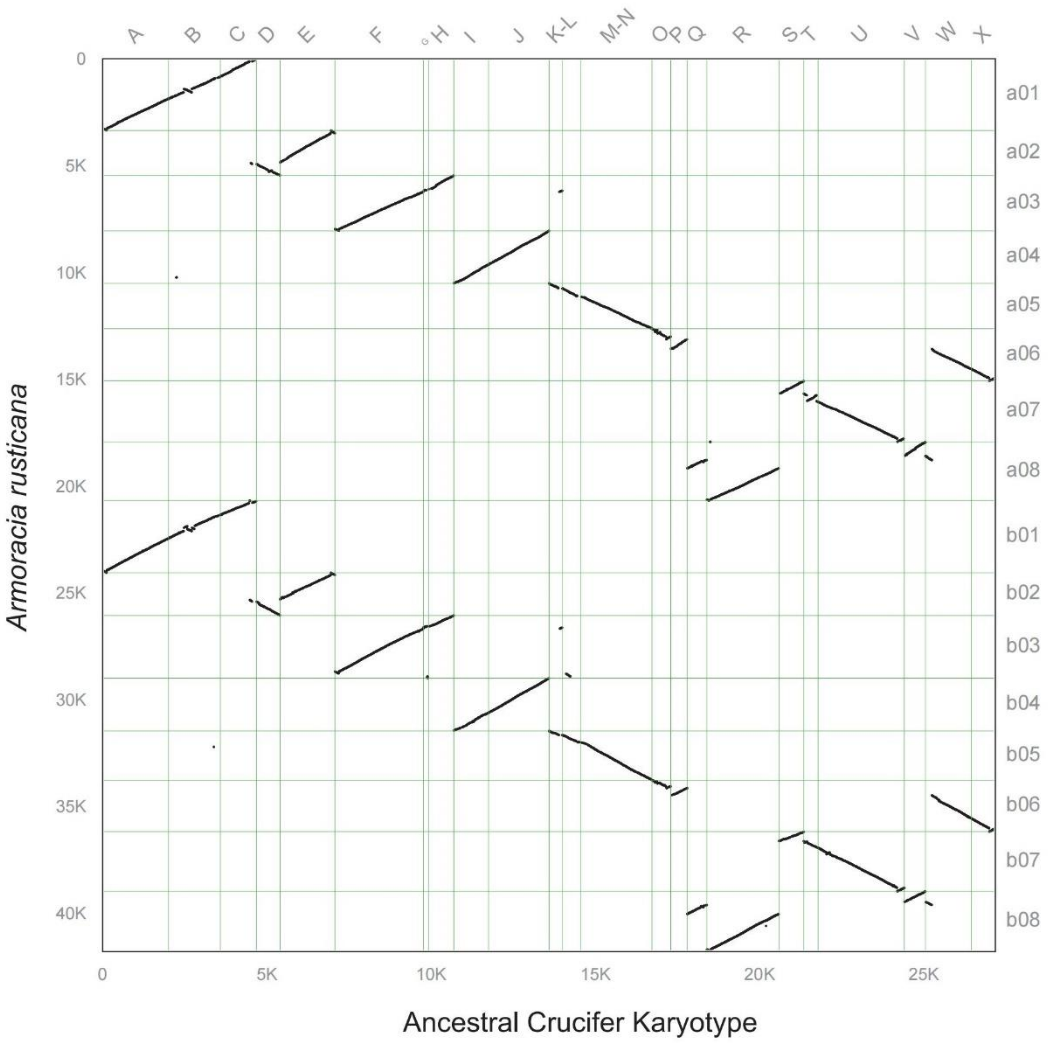

Supplementary Fig. 9. Pairwise synteny visualization of Ancestral Crucifer Karyotype and the telomere-to-telomere gap-free genome assembly of *Armoracia rusticana*.

A-X: The revised Ancestral Crucifer Karyotype (ACK) comprising 22 genomic blocks.

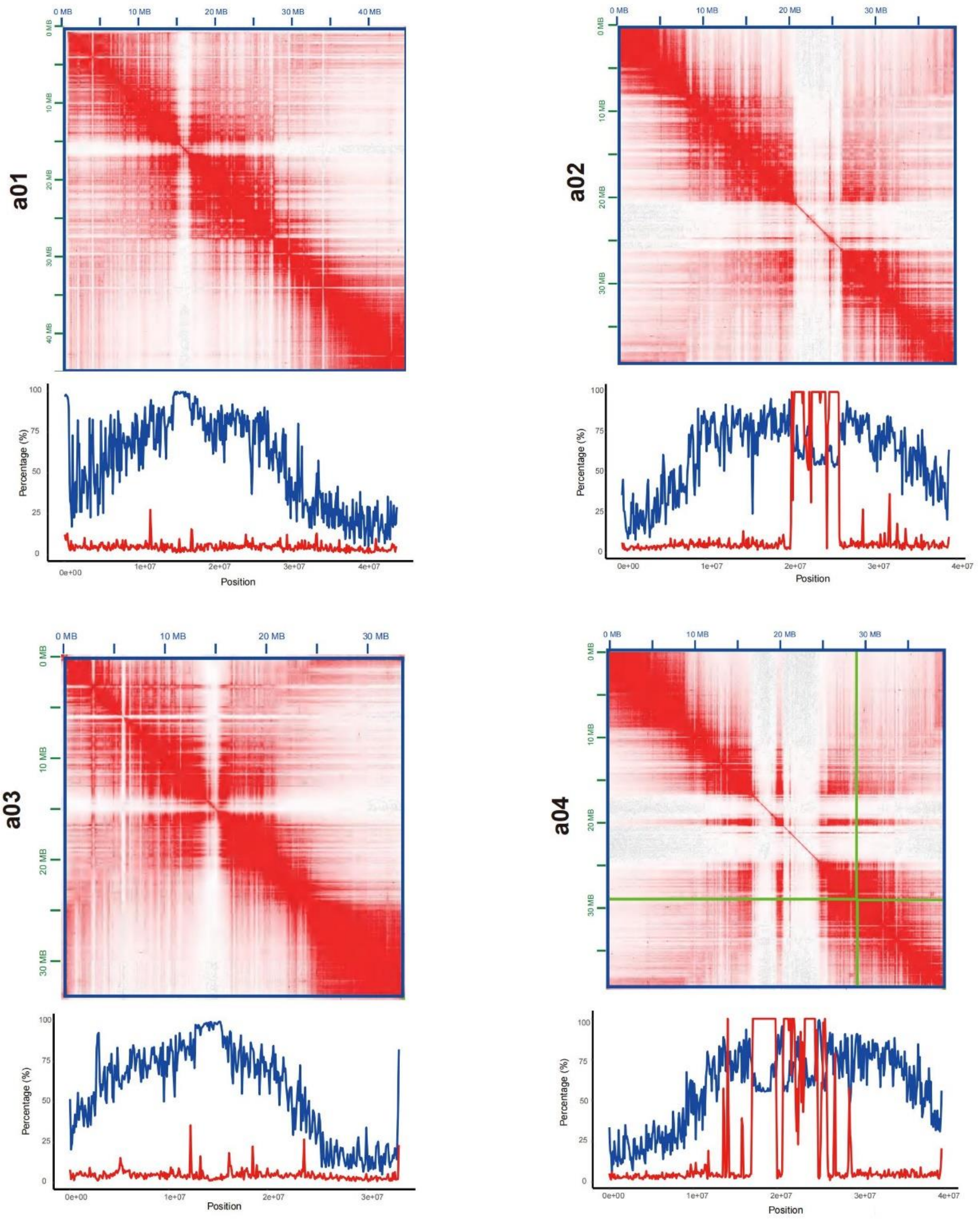

Supplementary Fig. 10. The Hi-C interaction heatmap and the density distribution of the repeats along the chromosome a01-a04.

The blue lines represent the density of the interspersed repeats within 100-kb sliding windows. The red lines represent the density of the tandem repeats within 100-kb sliding windows. The boundaries of contigs and scaffolds are indicated by green and blue lines, respectively. Source data are provided as a Source Data file.

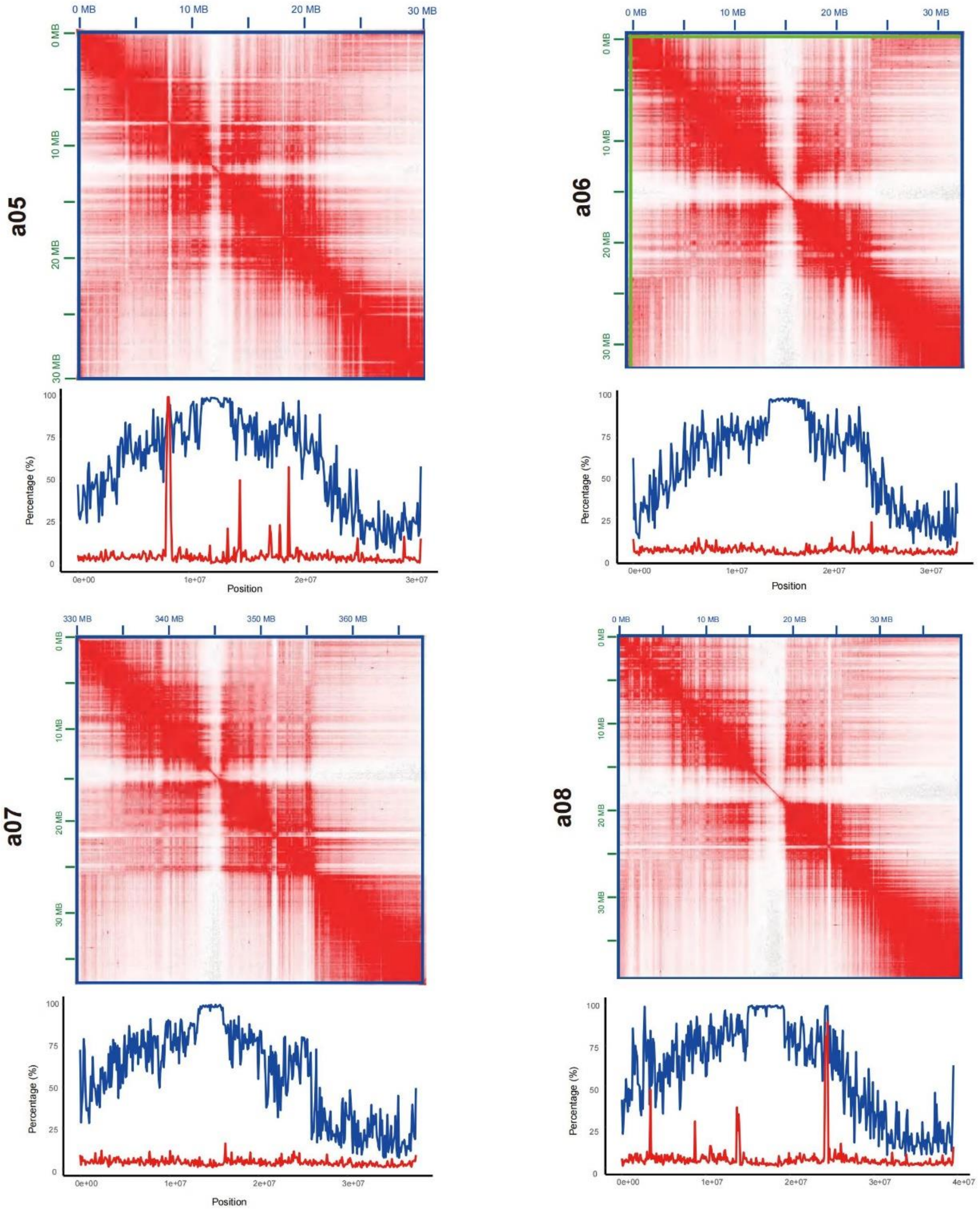

Supplementary Fig. 11. The Hi-C interaction heatmap and the density distribution of the repeats along the chromosome a05-a08.

The blue lines represent the density of the interspersed repeats within 100-kb sliding windows. The red lines represent the density of the tandem repeats within 100-kb sliding windows. The boundaries of contigs and scaffolds are indicated by green and blue lines, respectively. Source data are provided as a Source Data file.

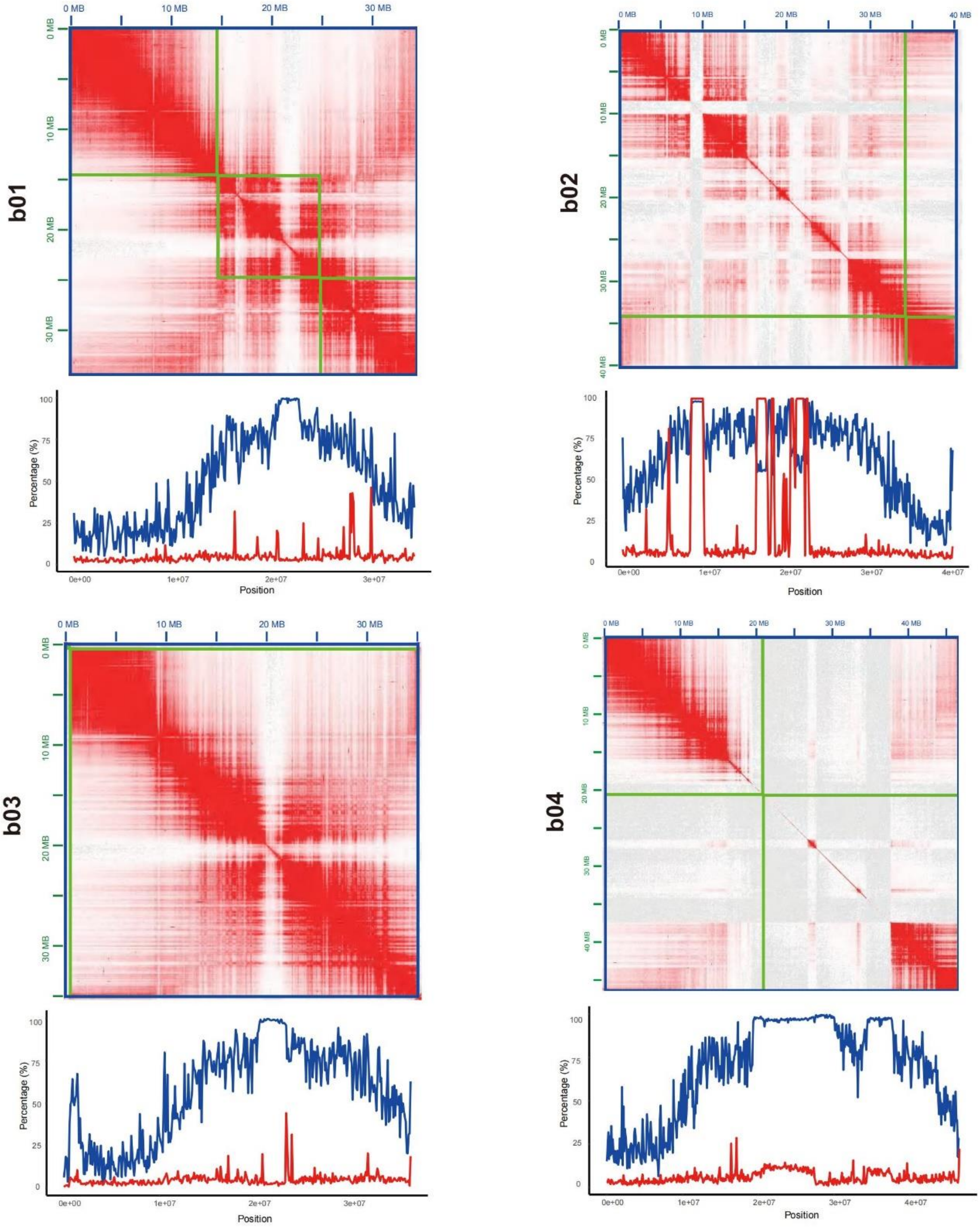

Supplementary Fig. 12. The Hi-C interaction heatmap and the density distribution of the repeats along the chromosome b01-b04.
The blue lines represent the density of the interspersed repeats within 100-kb sliding windows. The red lines represent the density of the tandem repeats within 100-kb sliding windows. The boundaries of contigs and scaffolds are indicated by green and blue lines, respectively. Source data are provided as a Source Data file.

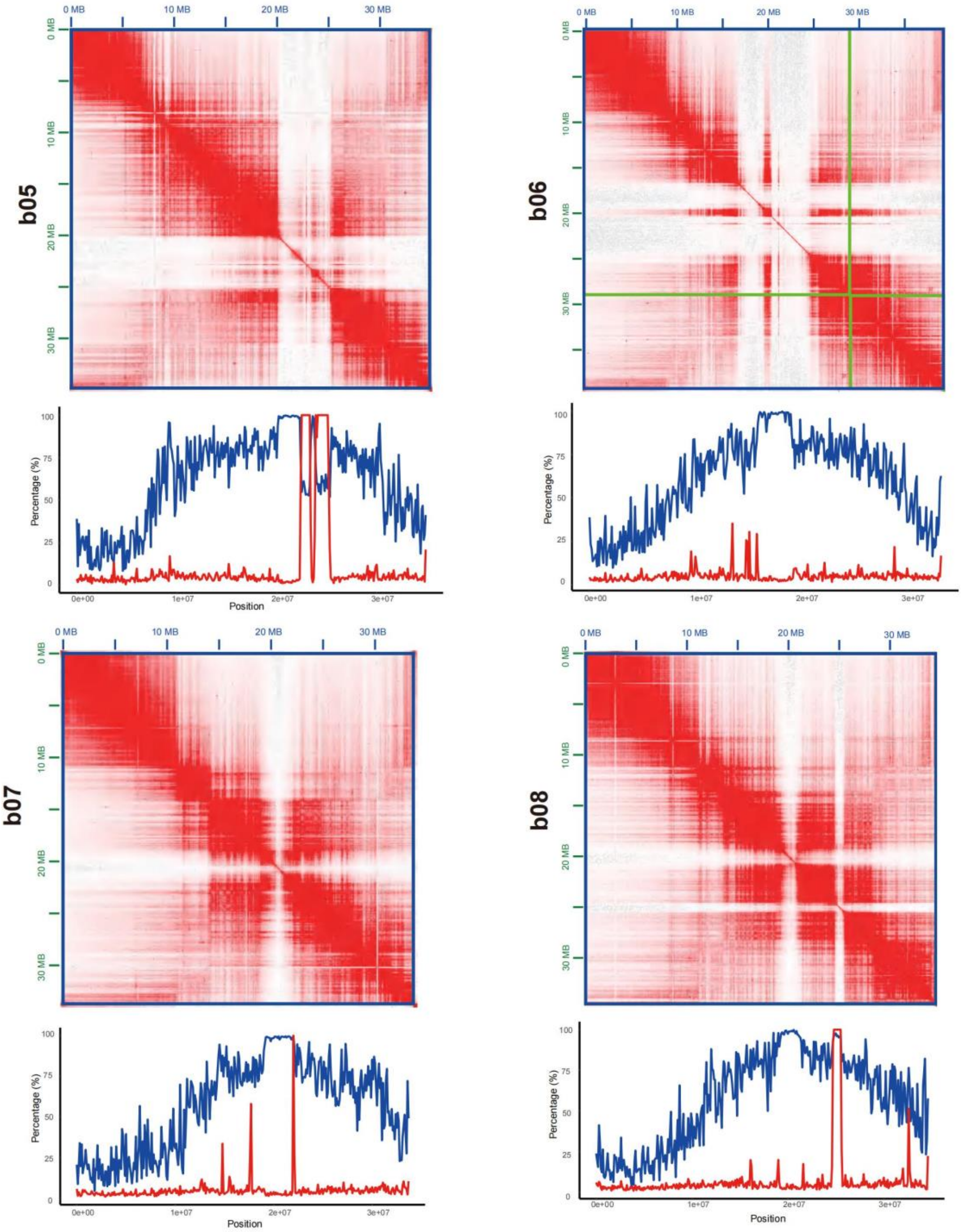

Supplementary Fig. 13. The Hi-C interaction heatmap and the density distribution of the repeats along the chromosome b05-b08.

The blue lines represent the density of the interspersed repeats within 100-kb sliding windows. The red lines represent the density of the tandem repeats within 100-kb sliding windows. The boundaries of contigs and scaffolds are indicated by green and blue lines, respectively. Source data are provided as a Source Data file.

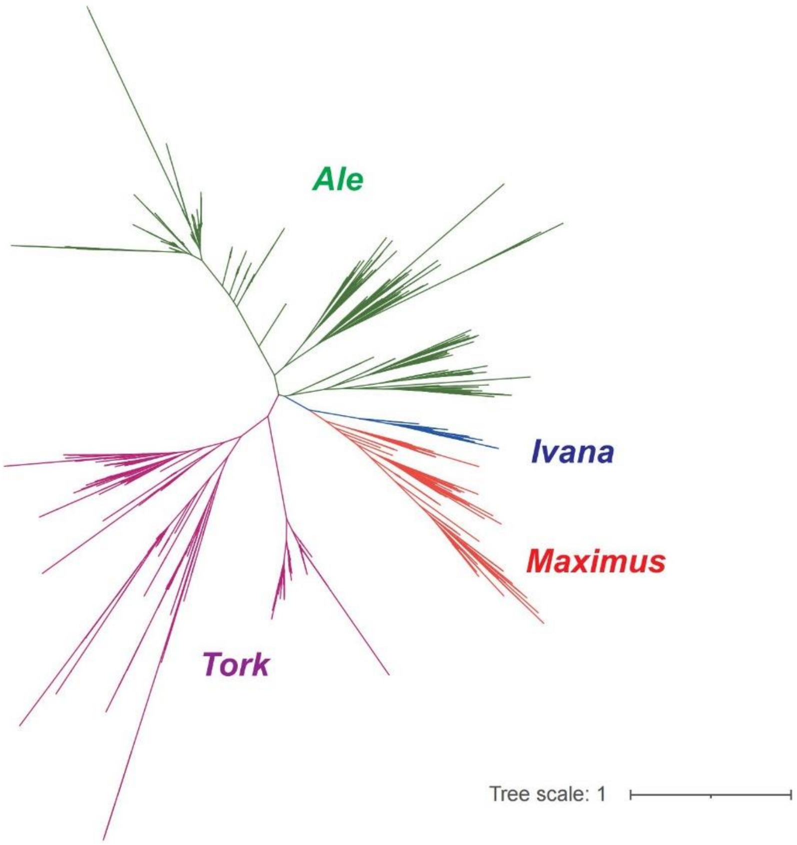

Supplementary Fig. 14. The phylogenetic tree of Ty1/*Copia* type LTR-RTs

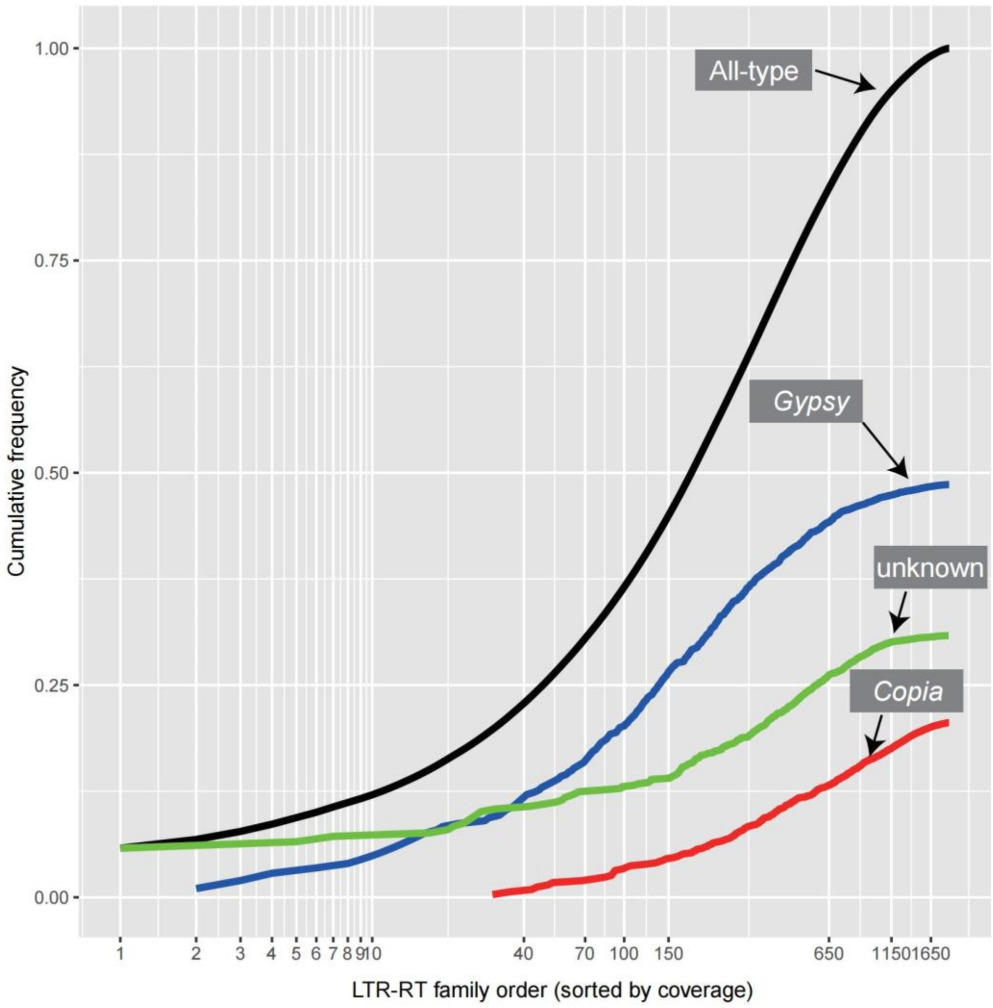

Supplementary Fig. 15. Cumulative frequency of the LTR-RT families in the sequenced horseradish genome.

Source data are provided as a Source Data file.

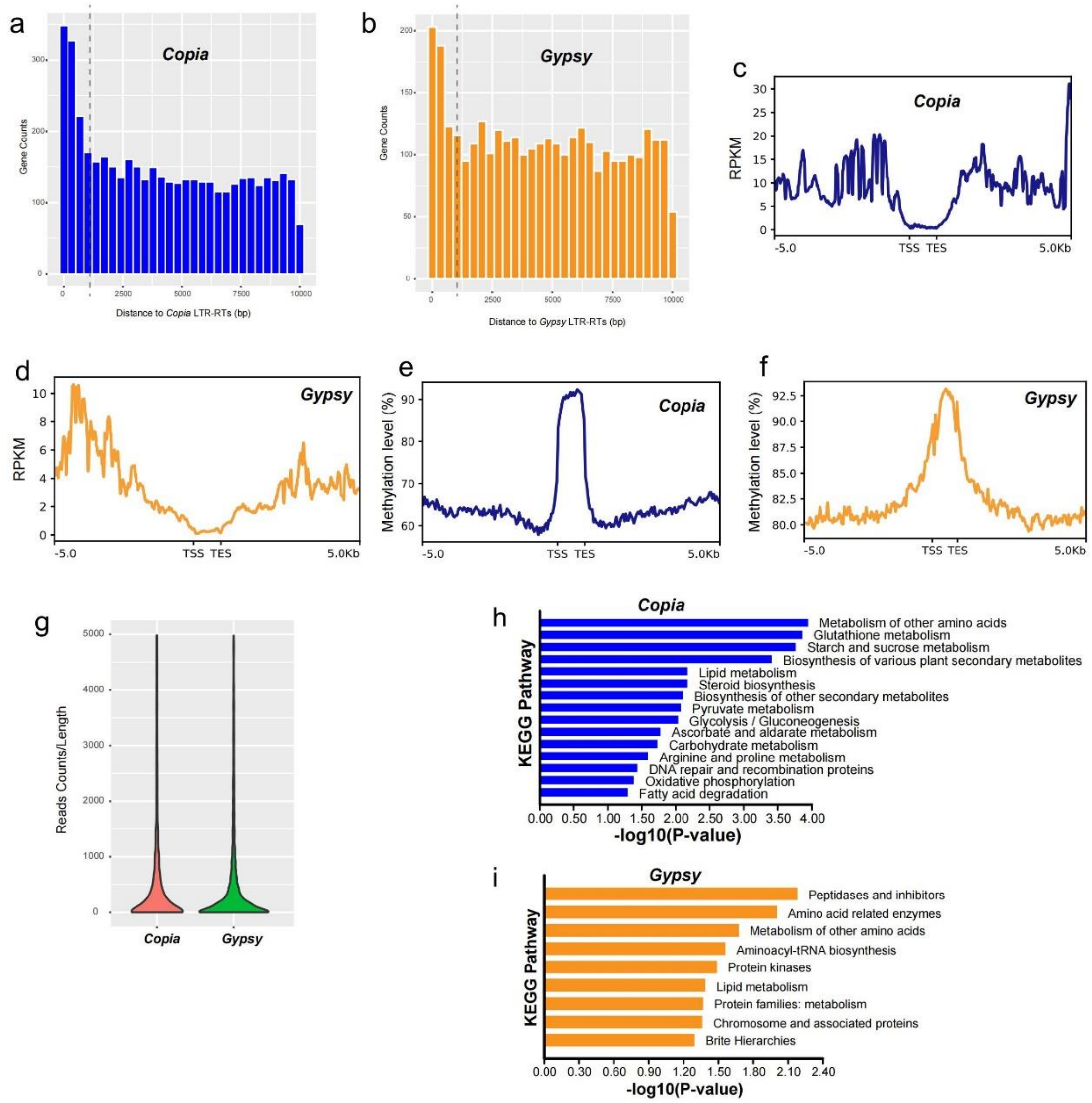

Supplementary Fig. 16. Characterization of long terminal repeat retrotransposons (LTR-RTs) and analysis of their nearby gene expression.

a-b, Statistics of the gene numbers near *Copia* (a) and *Gypsy* (b) type LTR-RTs； c-d, Distribution of expression levels near different types of LTR-RTs. The average expression level is based on values obtained by analysis of three different tissues (root, stem and leaf). The calculation was performed using deeptools, and the size of each sliding window was 40 bp. e-f, Distribution of methylation levels near different types of LTR-RTs. The methylation level was the average value of different tissues (root, stem, and leaf). g, Distribution of different types of LTR-RTs expression level. The average expression level is based on values obtained by analysis of three different tissues (root, stem, and leaf). The read count of each LTR-RT locus was obtained using the telescope software. We used the ratio of read counts and the length of LTR-RT to measure the expression level. h-i, The Kyoto Encyclopedia of Genes and Genomes (KEGG) enrichment analysis of the genes nearby different types of LTR-RTs. A one-sided Fisher’s exact test was adopted and adjustments were made for multiple comparisons with Benjamini and Hochberg method. FPKM: Fragments Per Kilobase of transcript per Million mapped reads. Source data are provided as a Source Data file.

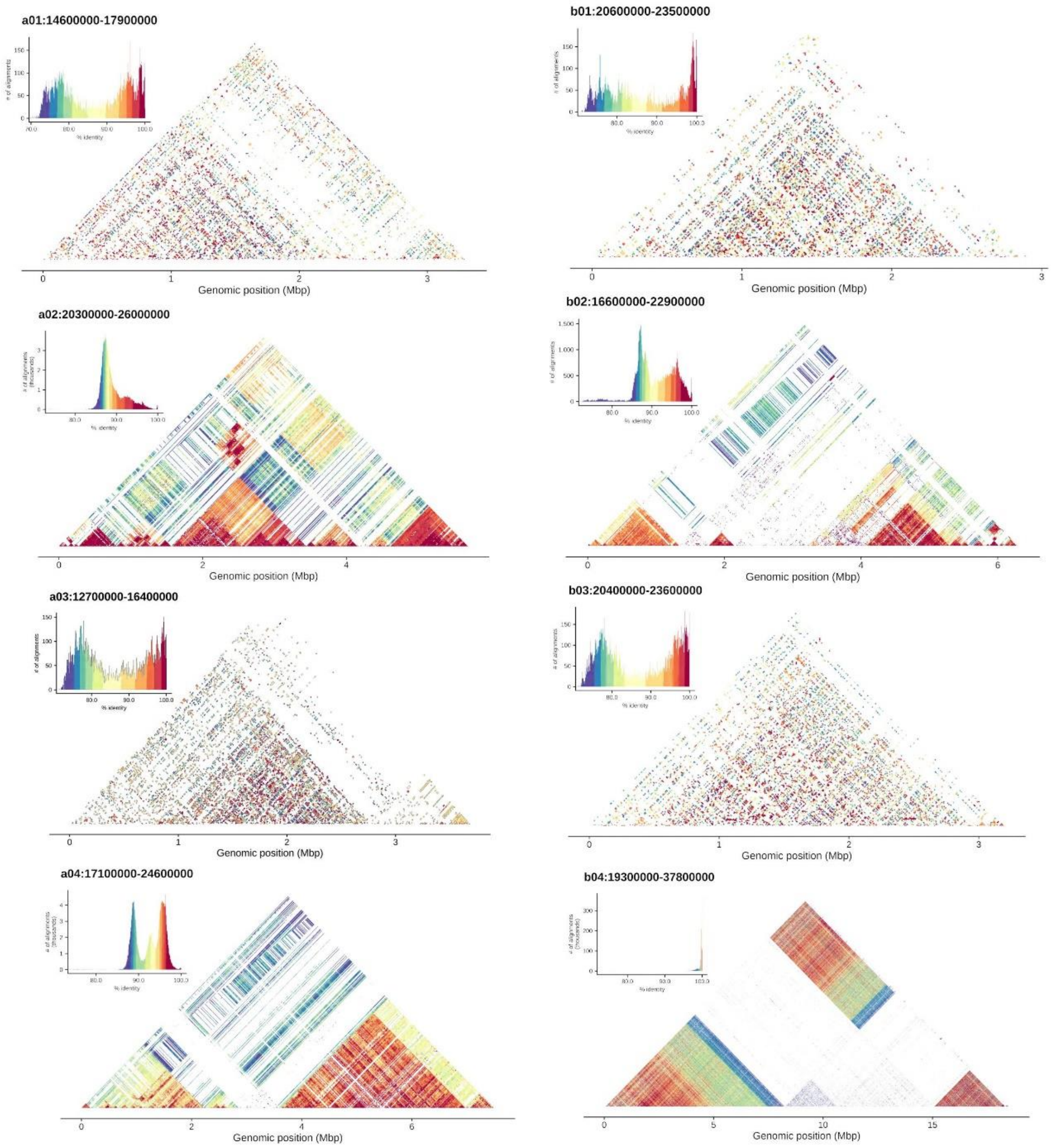

Supplementary Fig. 17. Structure of the centromeric regions in the horseradish genome (a01-a04 and b01-b04).

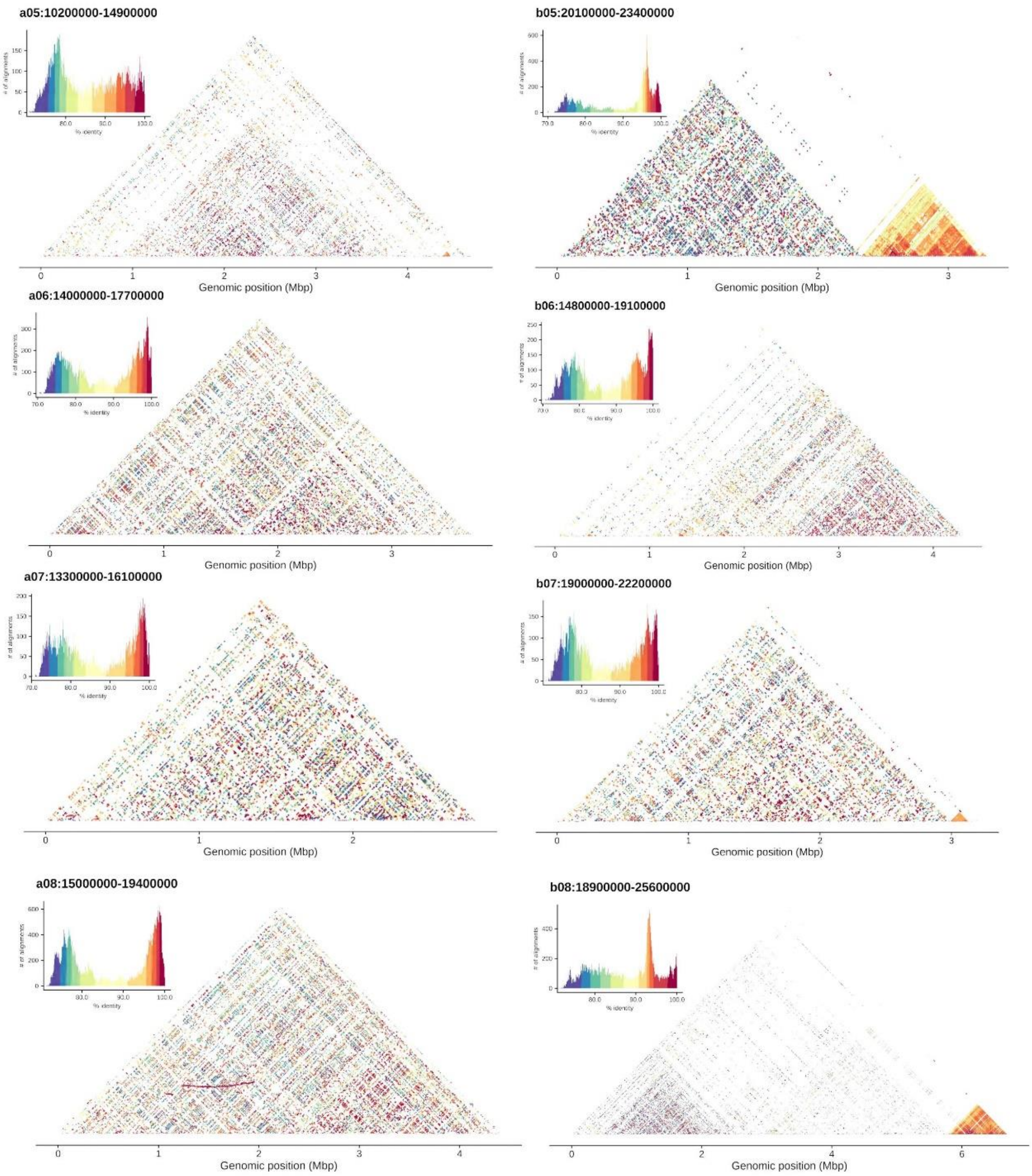

Supplementary Fig. 18. Structure of the centromeric regions in the horseradish genome (a05-a08 and b05-b08).

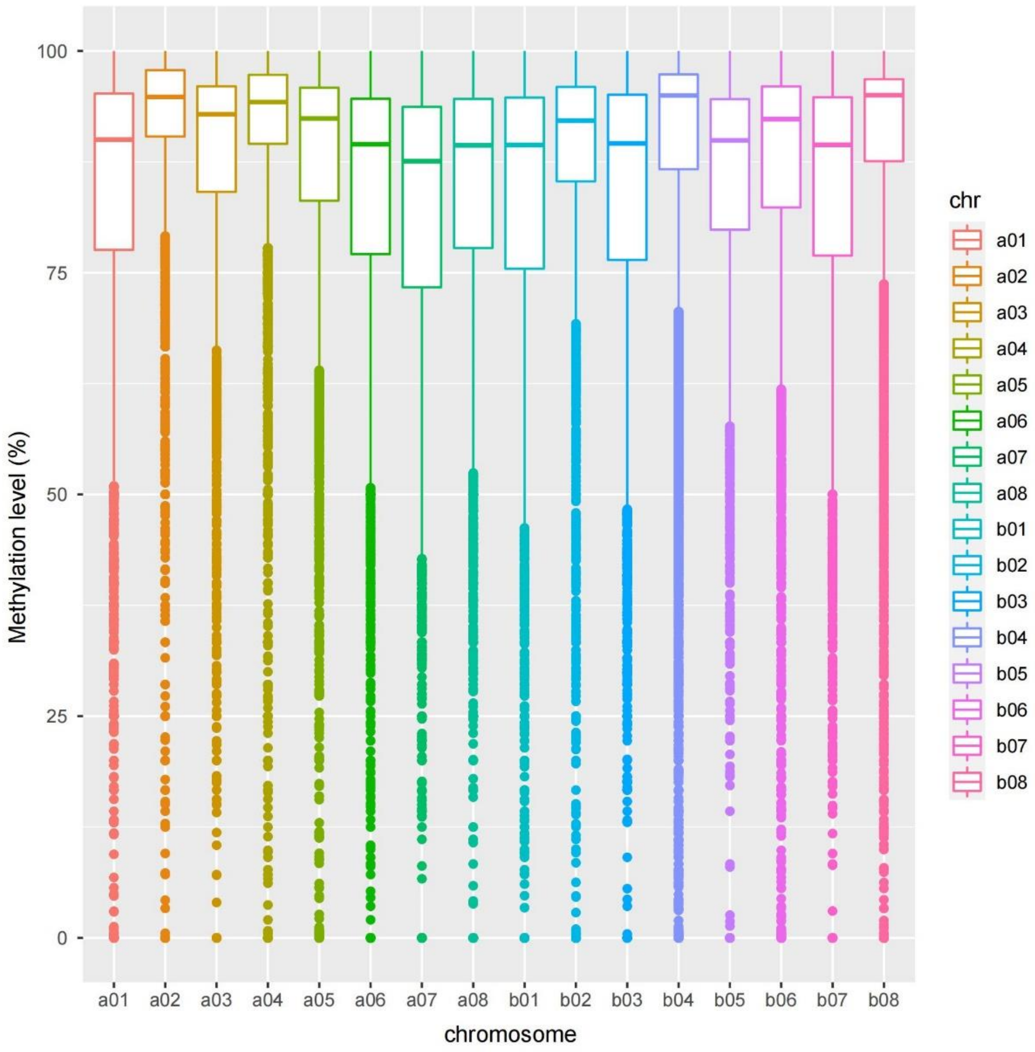

Supplementary Fig. 19. The methylation level of the centromeric regions in the horseradish genome.

The methylation level of centromeric regions was calculated using the sliding window strategy (with 100-kb sliding window). The weighted methylation level in each window was calculated and the averaged value was obtained across different tissues (root, stem and leaf). In the box plots, central lines: median values; box boundaries: 25th and 75th percentiles; whiskers: 1.5 * IQR (IQR: the interquartile range between the 25th and 75th percentile). Source data are provided as a Source Data file.

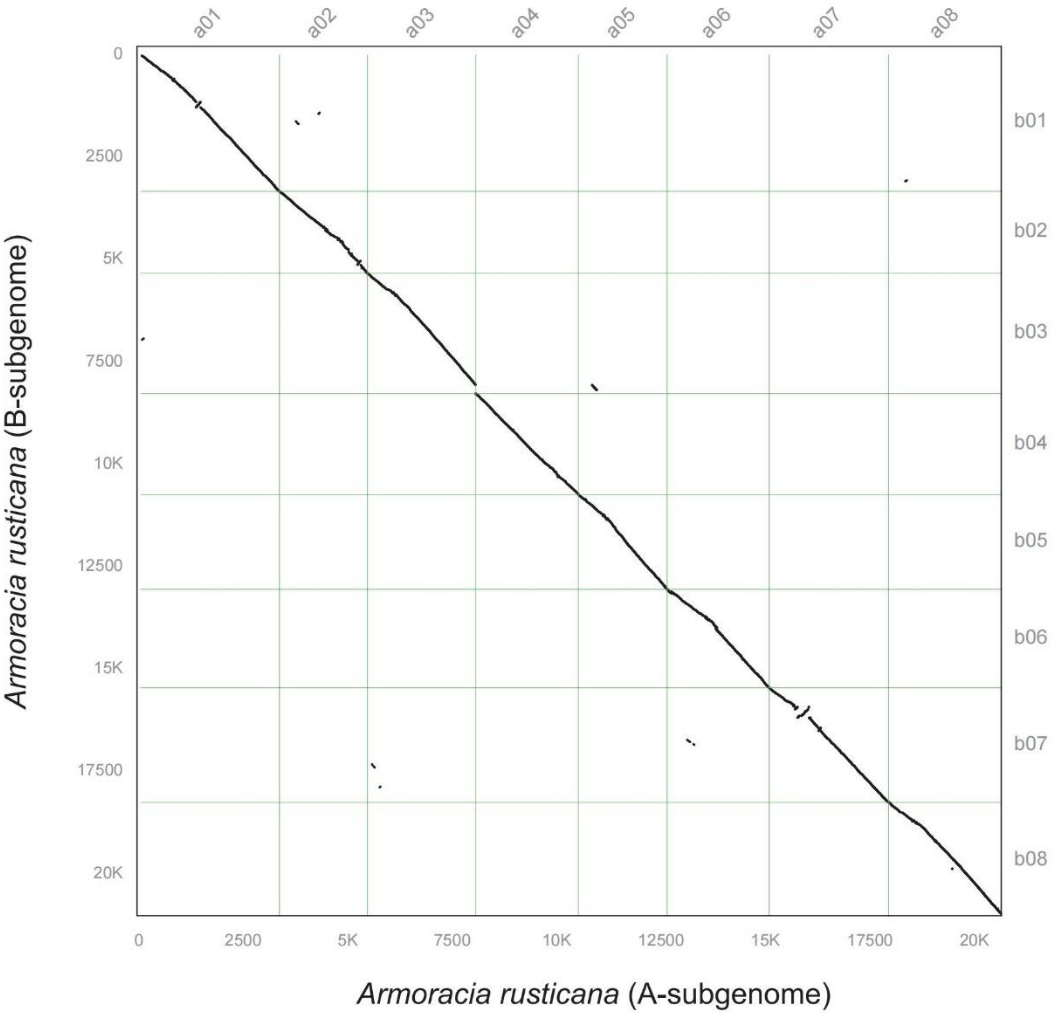

Supplementary Fig. 20. The visualization of pairwise synteny between horseradish subgenomes A and B.

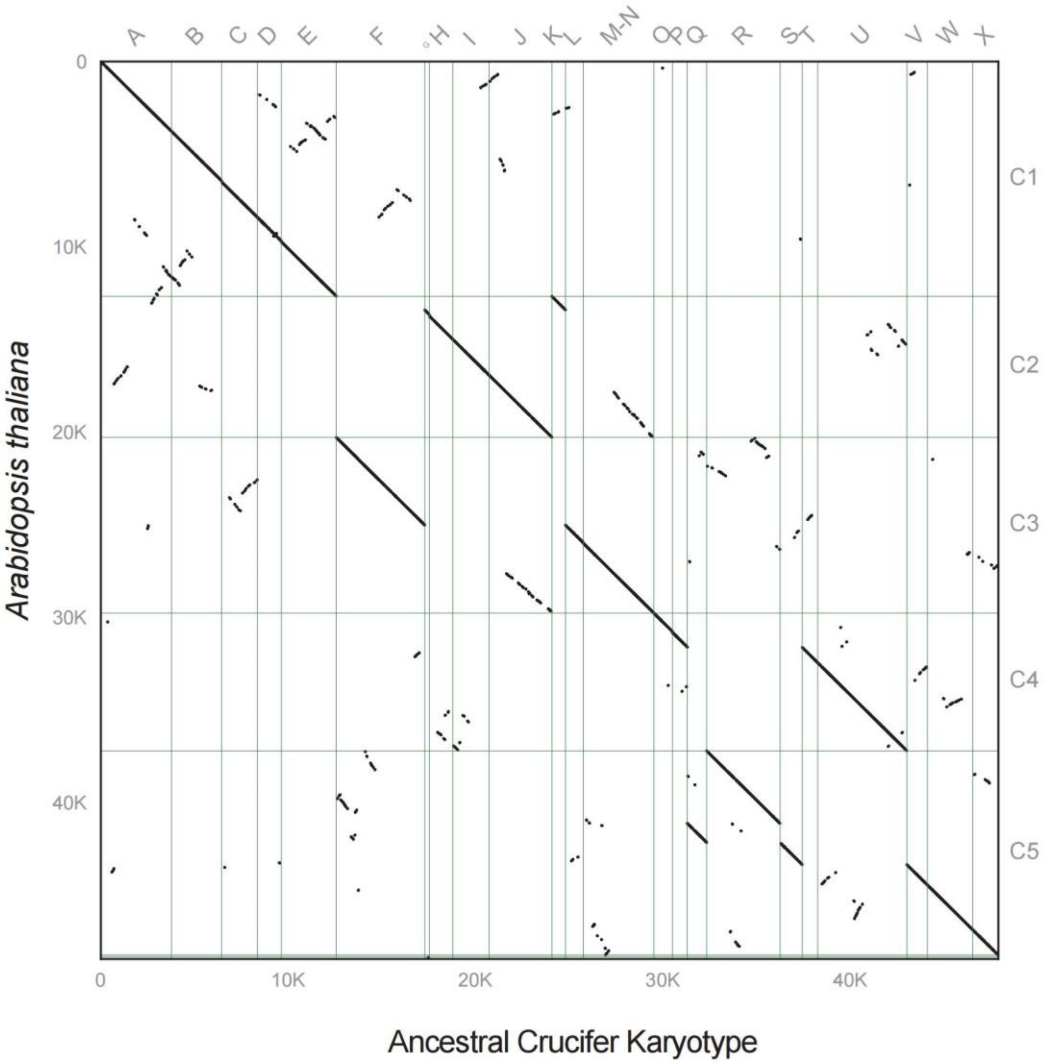

Supplementary Fig. 21. The visualization of pairwise synteny between 22 genomic blocks of the Ancestral Crucifer Karyotype and the five chromosomes of *Arabidopsis thaliana.*

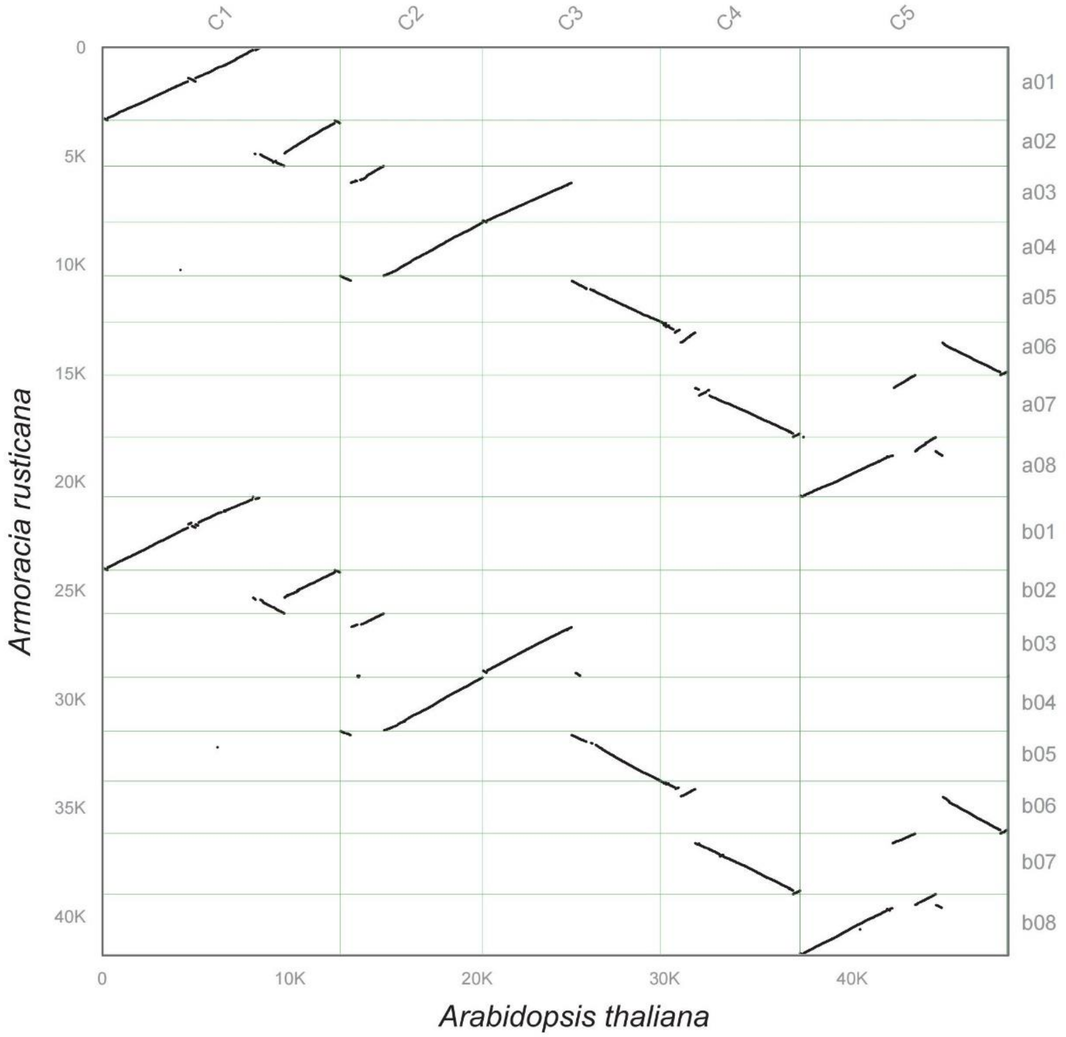

Supplementary Fig. 22. The visualization of pairwise synteny between 16 chromosomes of *A. rusticana* and five chromosomes of *Arabidopsis thaliana*.

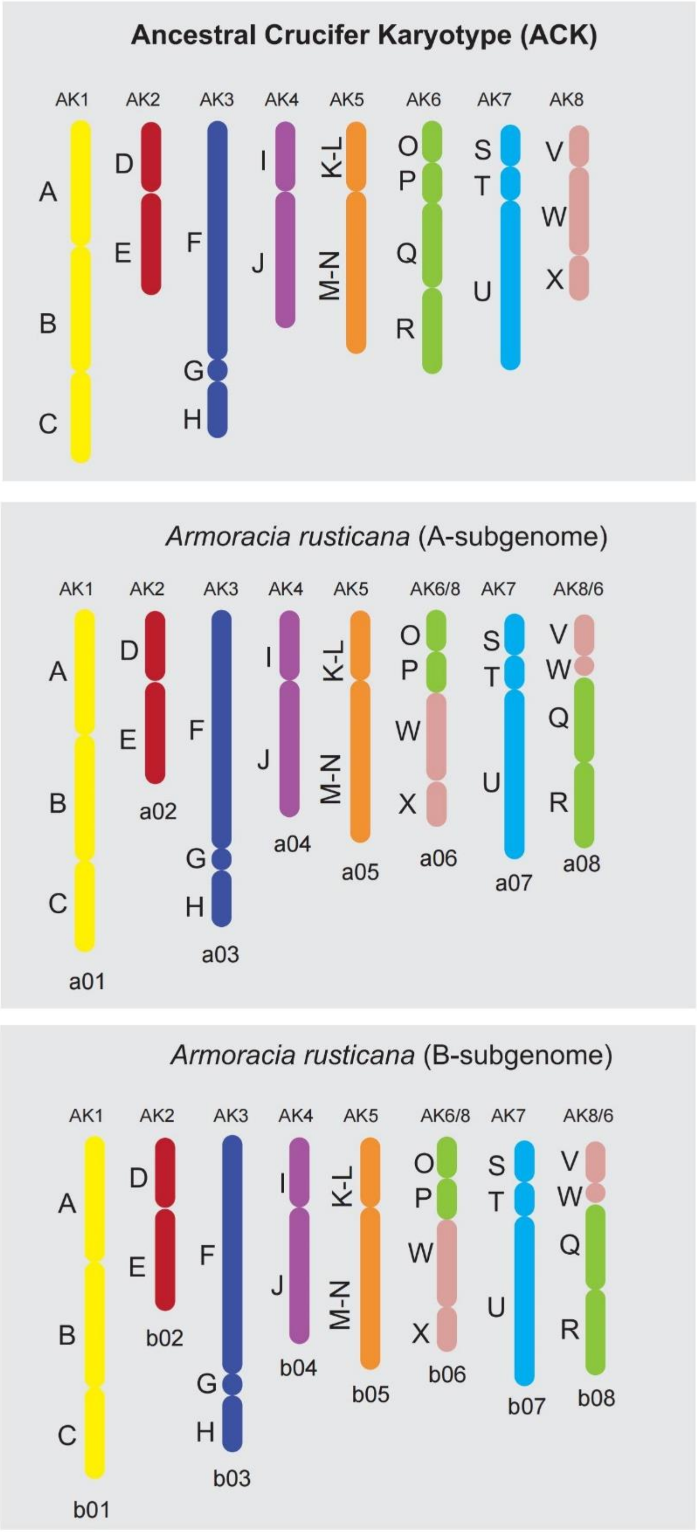

Supplementary Fig. 23. The Ancestral Crucifer Karyotype (ACK) and the inferred horseradish karyotype.

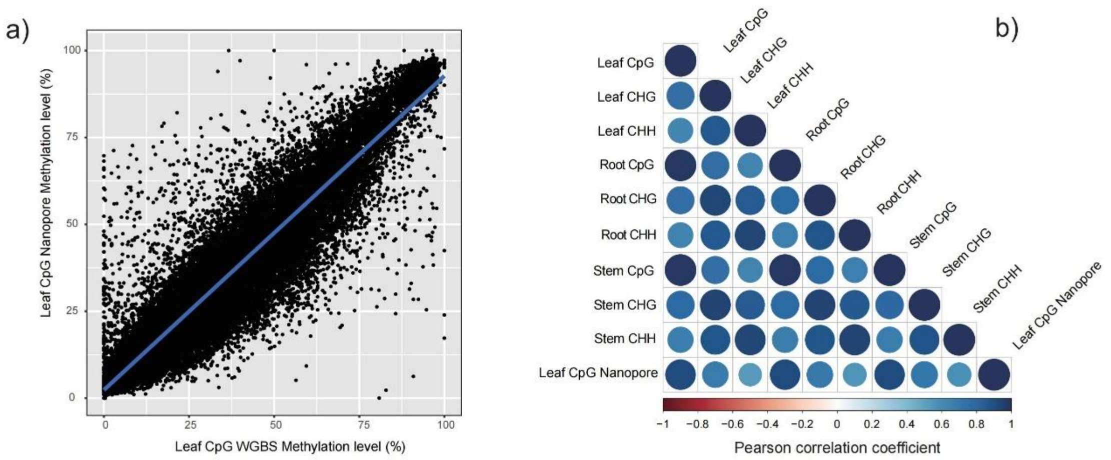

Supplementary Fig. 24. The correlation of the methylation level identified by Nanopore and whole genome bisulfite sequencing (WGBS) (a) and the correlation of the methylation levels in different tissues and cytosine contexts (b). Source data are provided as a Source Data file.

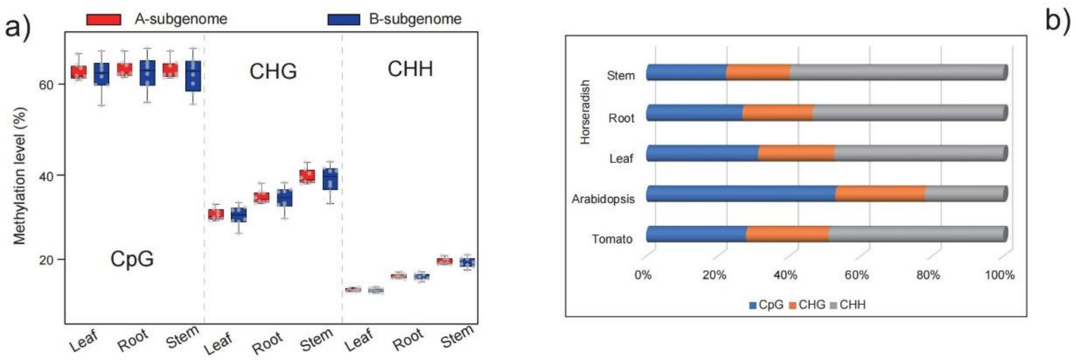

Supplementary Fig. 25. DNA methylation levels in different tissues (a) and percentage of the methylated cytosines in different tissues and sequence contexts (b).

a) The dots represent methylation levels of each chromosome of the two subgenomes in different sequence contexts (CpG, CHG and CHH) and tissues. In box plots, dots: data points; central lines: median values; box boundaries: 25th and 75th percentiles; whiskers: minimum to maximum.

b) The calculated percentage of methylated cytosines in different sequence contexts (CpG, CHG and CHH) and different tissues, compared with the values reported for *A. thaliana* and tomato[43].

Source data are provided as a Source Data file.

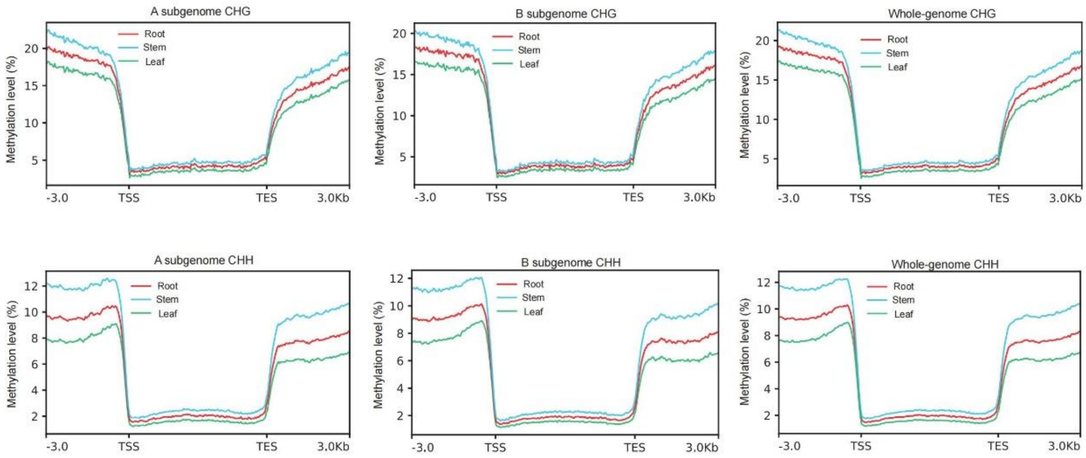

Supplementary Fig. 26. The methylation levels around the genes in different tissues and subgenomes.

Different cytosine contexts (CHG and CHH) were calculated using the whole genome bisulfite sequencing data.

TSS: transcription start site; TES: transcription end site.

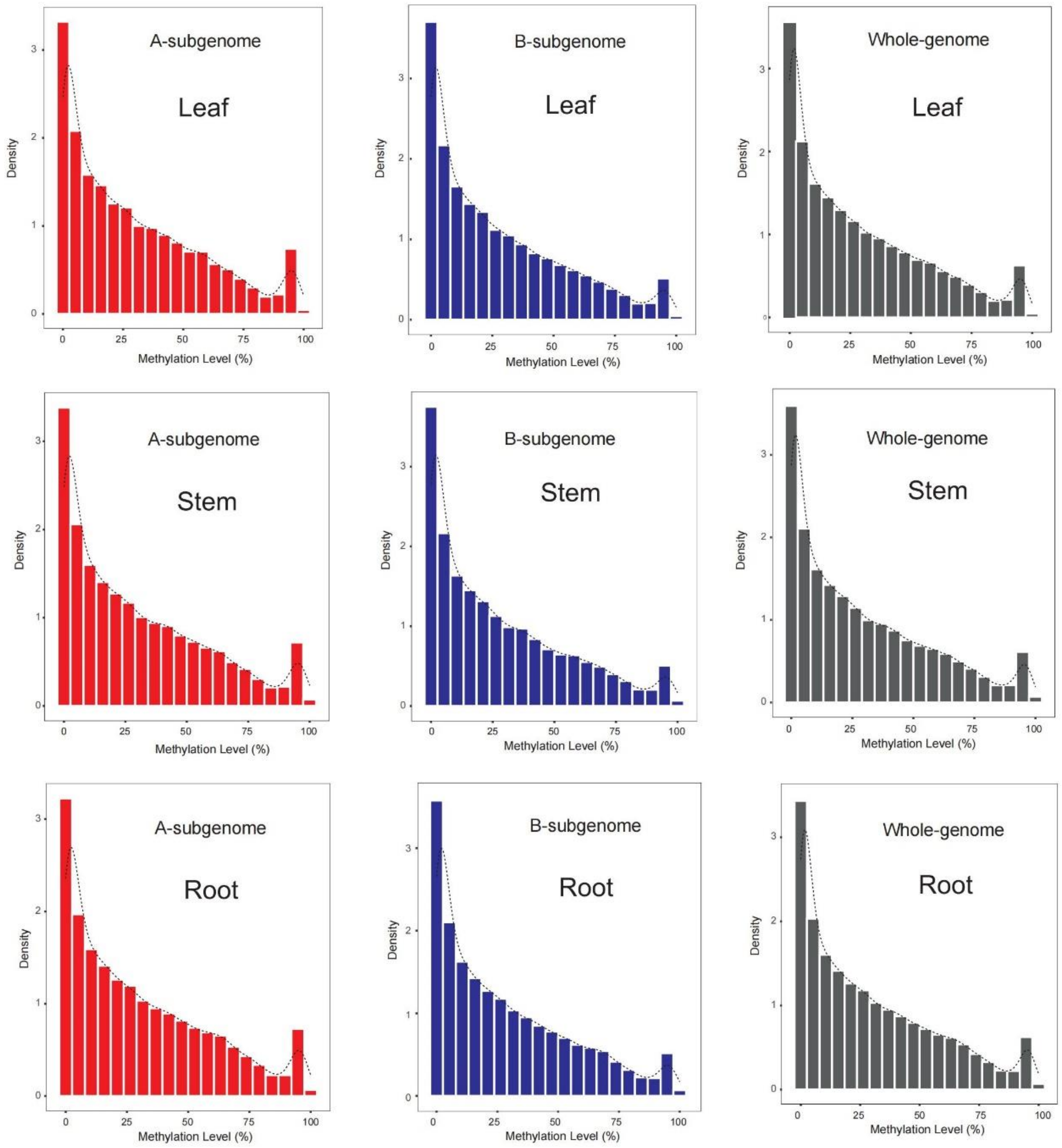

Supplementary Fig. 27. The distribution of methylation levels around the genes in different tissues and subgenomes.

The methylation levels were calculated using the whole genome bisulfite sequencing data. Source data are provided as a Source Data file.

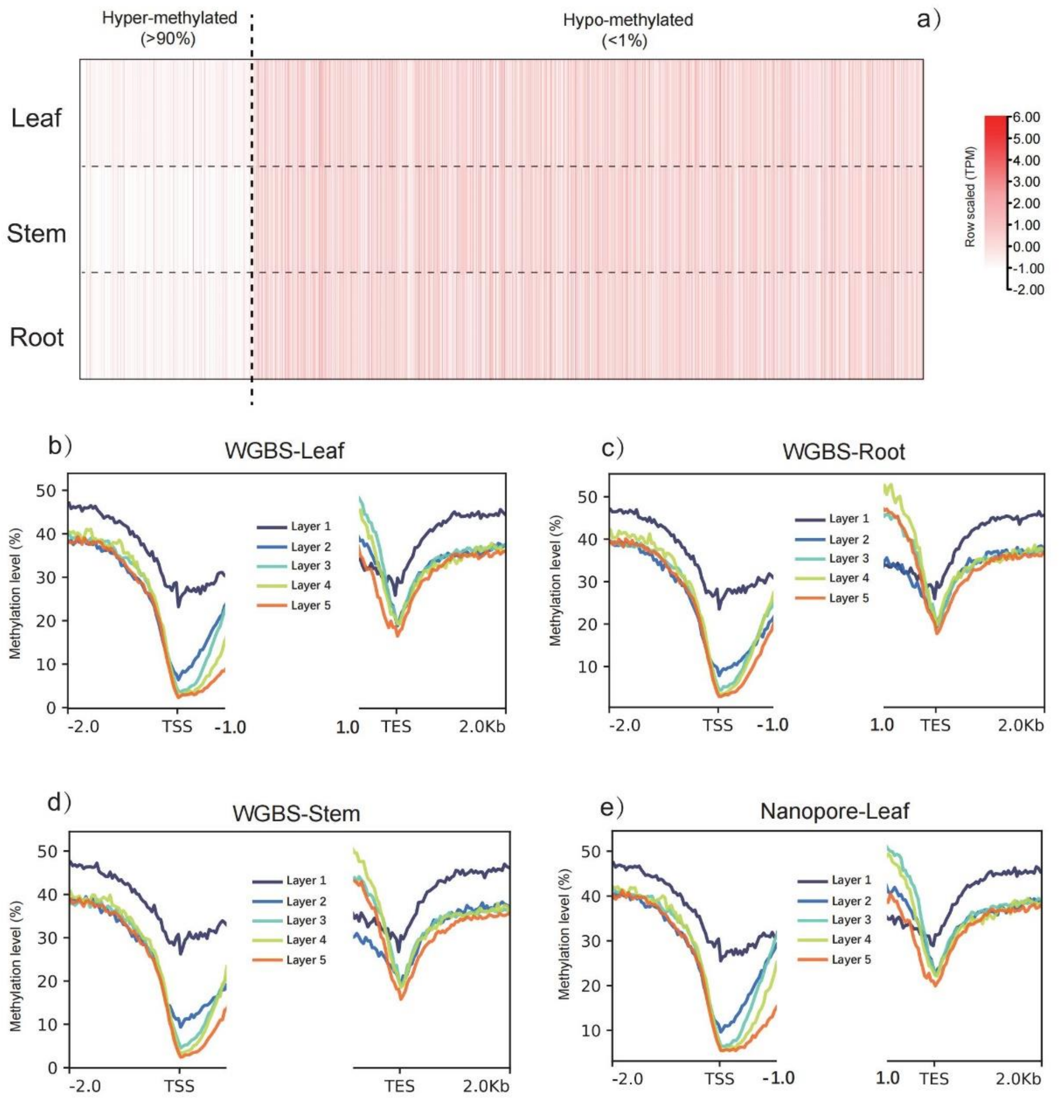

Supplementary Fig. 28. The correlation between the methylation levels around the genes and the gene expression levels.

a) The gene expression heatmap of hyper-methylated (methylation level >90%) and hypo-methylated (methylation level <1%) genes. b-e) The CpG methylation levels around the genes in different tissues. The genes were divided into five expression layers (Layer1: TPM >=0 & <1; Layer 2: TPM >=1 & <5; Layer3: TPM >=5 & <10; Layer4: TPM>=10 <30; Layer5: TPM>=30). The methylation levels were calculated using whole genome bisulfite sequencing (WGBS) and Nanopore sequencing data, respectively.

Source data are provided as a Source Data file.

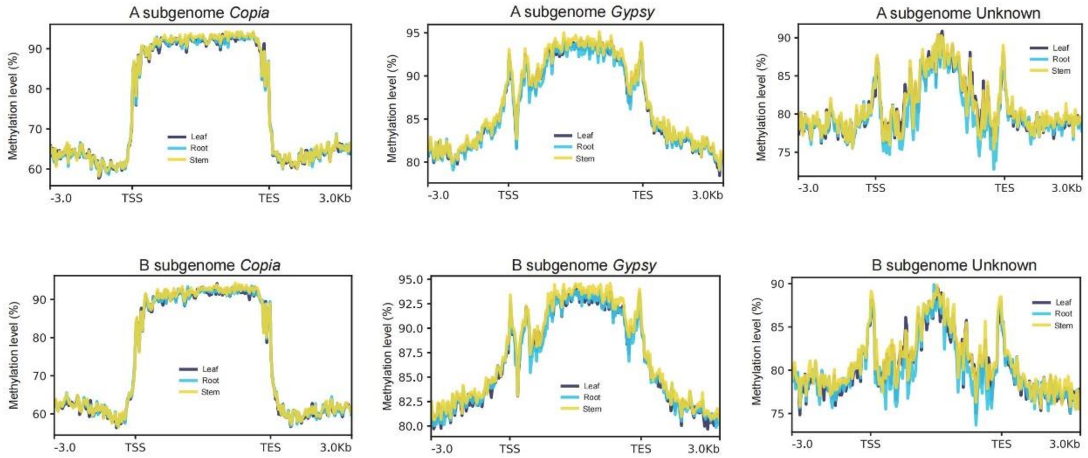

Supplementary Fig. 29. The methylation levels (CpG) around different types of LTR retrotransposons within both subgenomes and in different tissues.

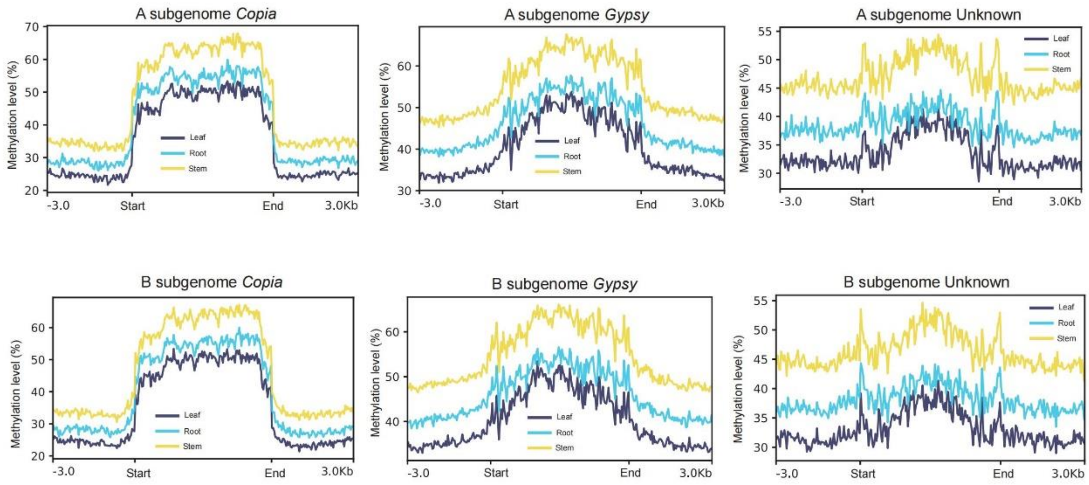

Supplementary Fig. 30. The methylation levels (CHG) around different types of LTR retrotransposons within both subgenomes and in different tissues.

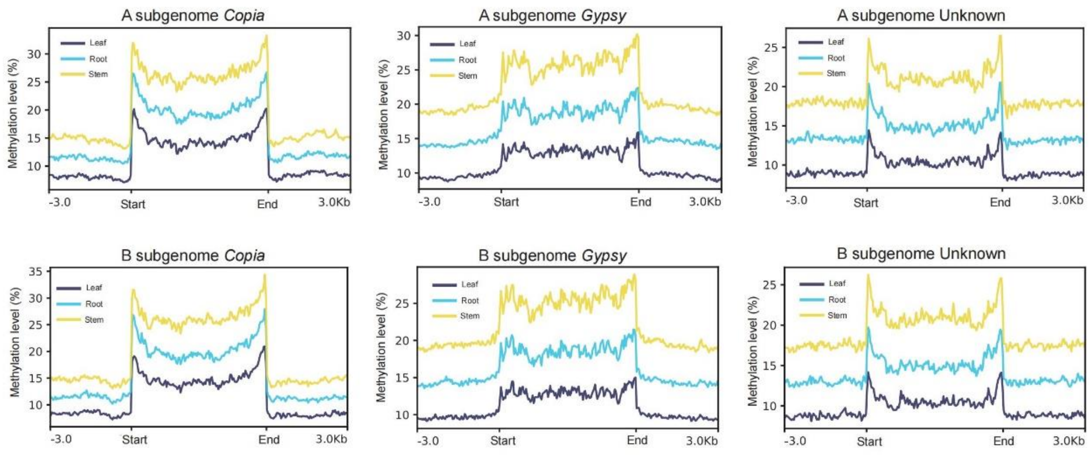

Supplementary Fig. 31. The methylation levels (CHH) around different types of LTR retrotransposons within both subgenomes and in different tissues.

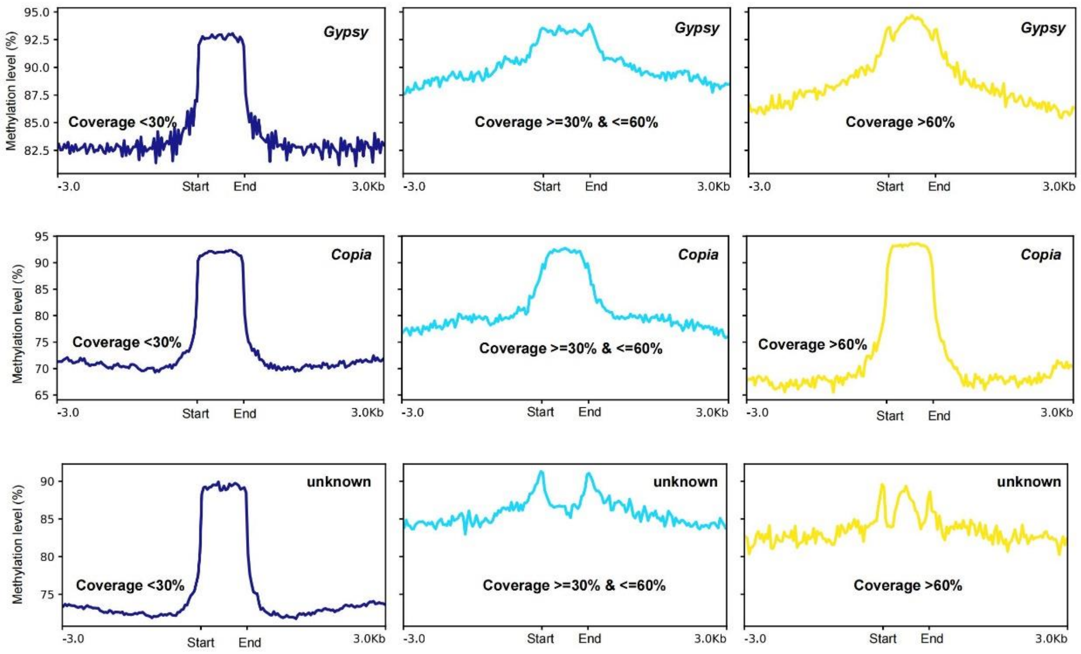

Supplementary Fig. 32. The methylation levels (CpG) around different types of LTR retrotransposon fragments.

Fragmented LTR retrotransposons (LTR-RTs) were annotated with RepeatMasker using the sequences of intact LTR retrotransposons as the library. The alignment coverage (the length of fragmented LTR retrotransposons/the length of intact LTR-RTs) obtained from the output of RepeatMasker. Fragmented LTR-RTs were classified into three classifications based on their alignment coverage. The methylation levels around different types of LTR-RT fragments were profiled using Deeptools.

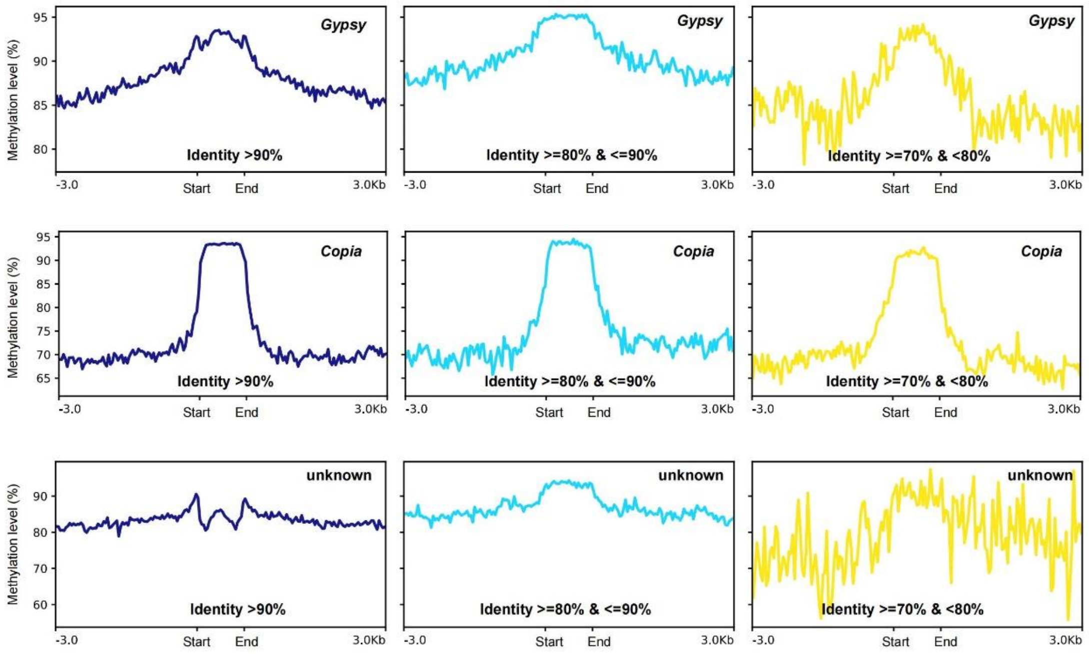

Supplementary Fig. 33. The methylation levels (CpG) around different types of LTR retrotransposon fragments (coverage >50%).

Fragmented LTR retrotransposons (LTR-RTs) were annotated with RepeatMasker using the sequences of intact LTR-RTs the library. The alignment identity between intact LTR-RTs and identified fragmented LTR-RTs was obtained from the output of Repeatmasker. The fragmented LTR-RTs (alignment coverage >50%) were classified into three classifications based on their alignment identity. The methylation levels around different LTR-RT fragments were profiled using Deeptools.

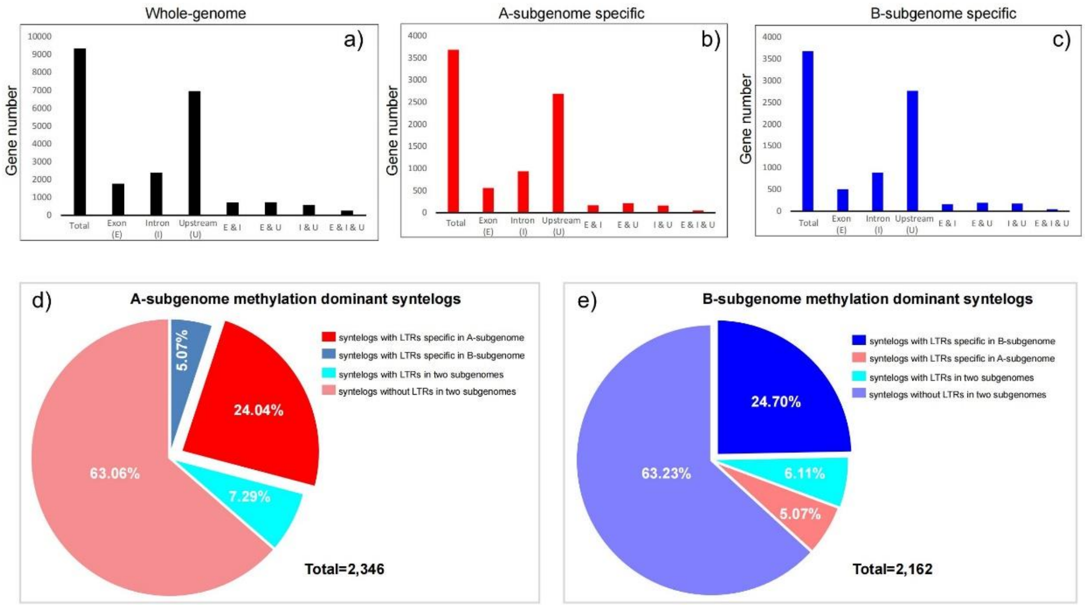

Supplementary Fig. 34. The relationship between differentially methylated gene pairs and fragmented LTR retrotransposons.

a) The statistics of the genes haboring LTR-RT fragments in exon, intron or upstream region (-500 bp). b) The statistics of the A-subgenome genes haboring LTR-RT fragments in exon, intron or upstream region. c) The statistics of the B-subgenome genes haboring LTR-RT fragments in exon, intron or upstream region. d) The distribution of the LTR-RT fragments in the differentially methylated gene pairs that exhibited higher methylation level in the A-subgenome. e) The distribution of the LTR fragments in the differentially methylated gene pairs that exhibited higher methylation level in the B-subgenome.

Source data are provided as a Source Data file.

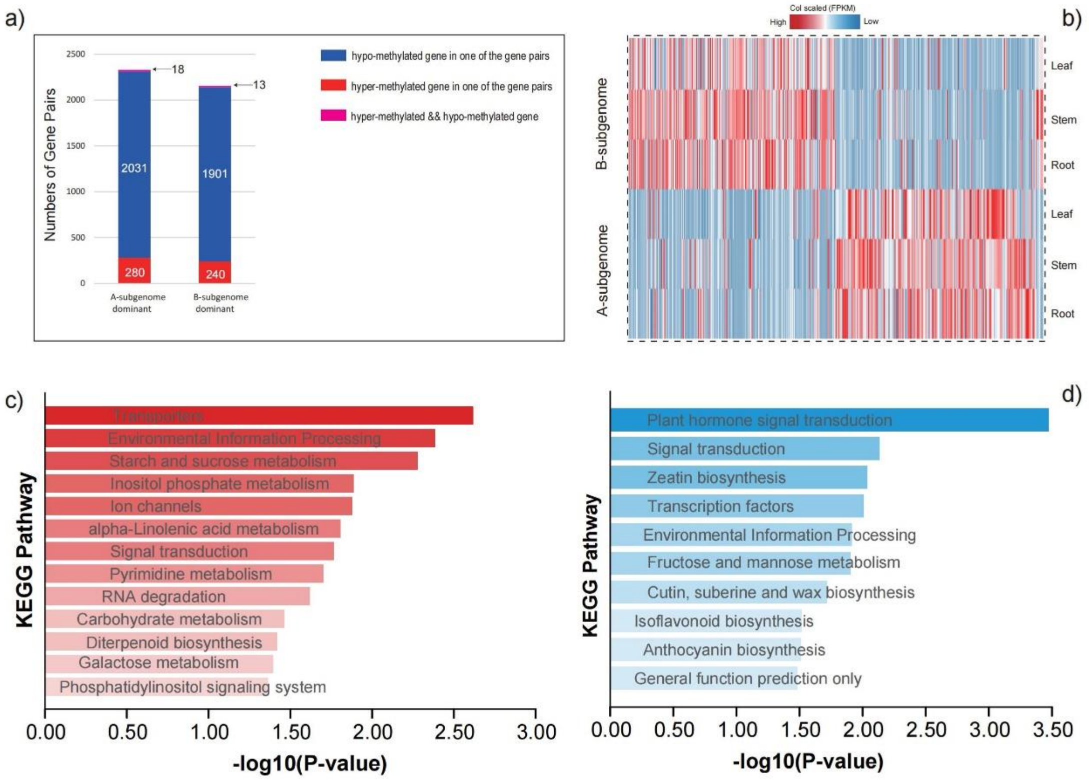

Supplementary Fig. 35. The statistics of the differentially methylated gene pairs between two subgenomes of horseradish.

a) The numbers of the differentially methylated genes dominant (with significantly greater methylation levels) in A and B subgenomes. We defined a gene pair as differentially methylated if at least one gene belonged to the hyper- or hypo-methylated genes, along with a ratio of methylation level greater than 2. The gene pairs were further classified into three groups: (1) one hypo-methylated gene (methylation level <1%) of a gene pair; (2) one hyper-methylated gene (methylation level >90%) of a gene pair; (3) one hyper-methylated and one hypo-methylated gene of a gene pair.

b) The expression heatmap of the differentially methylated gene pairs.

c-d) The KEGG (Kyoto Encyclopedia of Genes and Genomes) pathway enrichment analysis of differentially methylated genes dominant (with greater methylation levels) in A and B subgenome.

A one-sided Fisher's exact test was adopted and adjustments were made for multiple comparisons with Benjamini and Hochberg method. Source data are provided as a Source Data file.

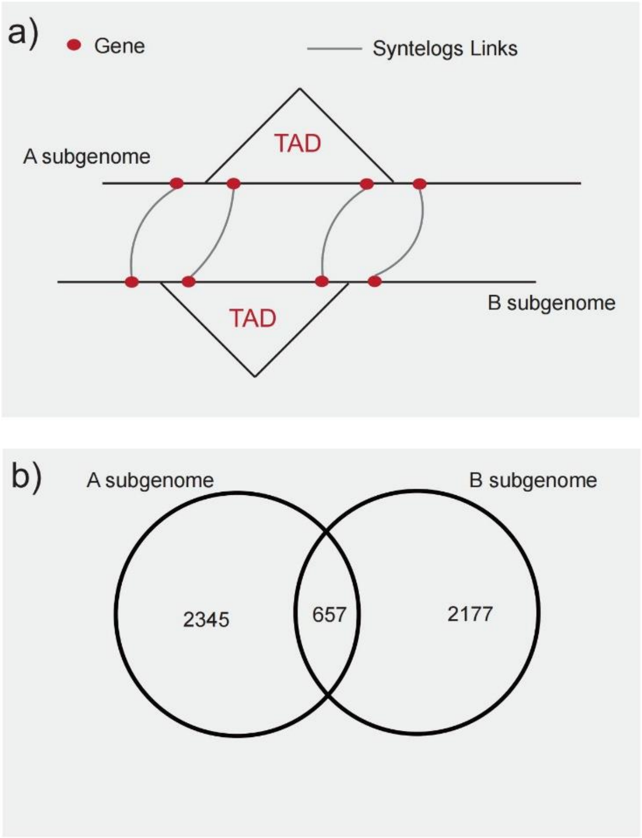

Supplementary Fig. 36. The topologically associated domains (TAD) diversification between two subgeomes.

a) The schematic graph of the comparison of TAD boundaries between two subgenomes. The four genes nearby the TAD boundaries were extracted and defined as TAD-associated genes (TAD-g). Within the syntenic blocks, the TAD-g in syntelogs can reflect the diversification of TAD boundaries between two subgenomes.

b) Veengraph of the TAD-g in syntelogs of each subgenome. The overlapped region indicates the TAD-g in syntelogs.

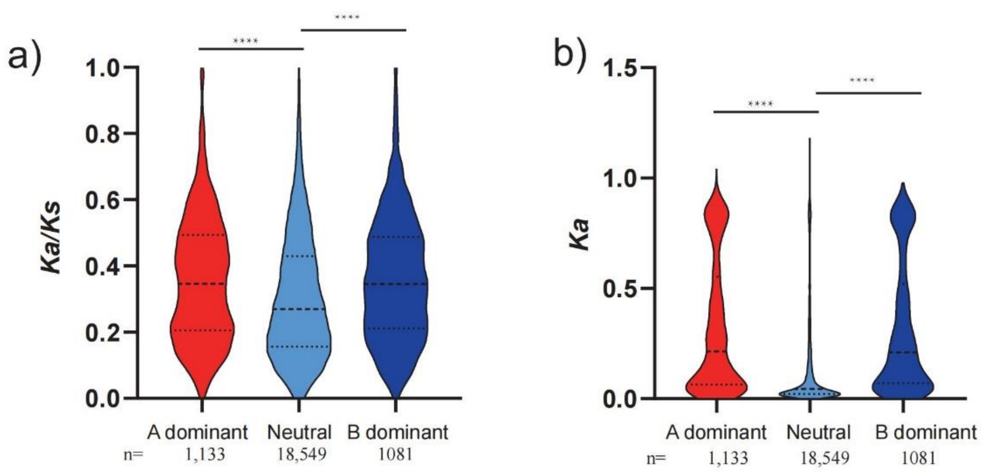

Supplementary Fig. 37. The *Ka/Ks* (a) and *Ka* (b) of the subgenome A/B dominant genes and neutrally expressed genes.

The number (n) of data points for each violin is shown below. In the violin plots, central line: median values; other two horizonal lines: 25th and 75th percentiles. Significance was tested with two-sided Mann Whitney U test. ****, $P < 0.0001$. Source data are provided as a Source Data file.

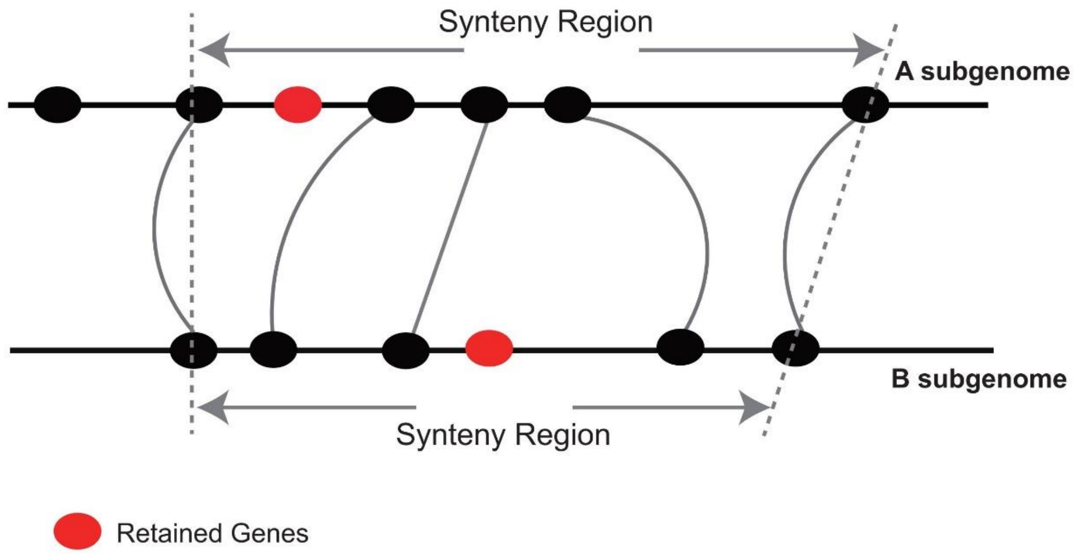

Supplementary Fig. 38. Schematic diagram of the method to detect gene losses in A and B subgenomes of horseradish.

The red ball indicates retained genes in the B subgenome and their syntelogs in the A subgenome were lost.

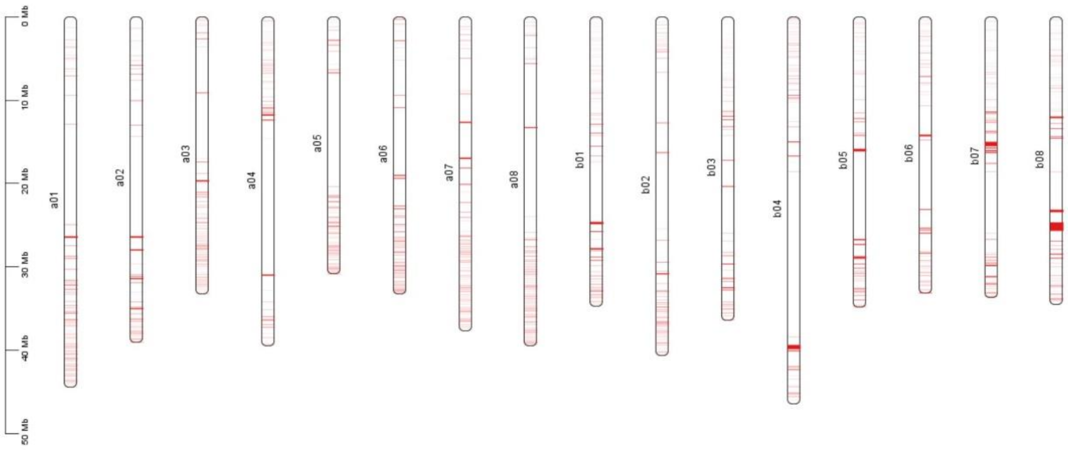

Supplementary Fig. 39. The genomic position of the predicted lost genes in each chromosome of horseradish.

The lost genes in each chromosome were identified by comparing them with their homoeologous chromosome in the counterpart subgenome.

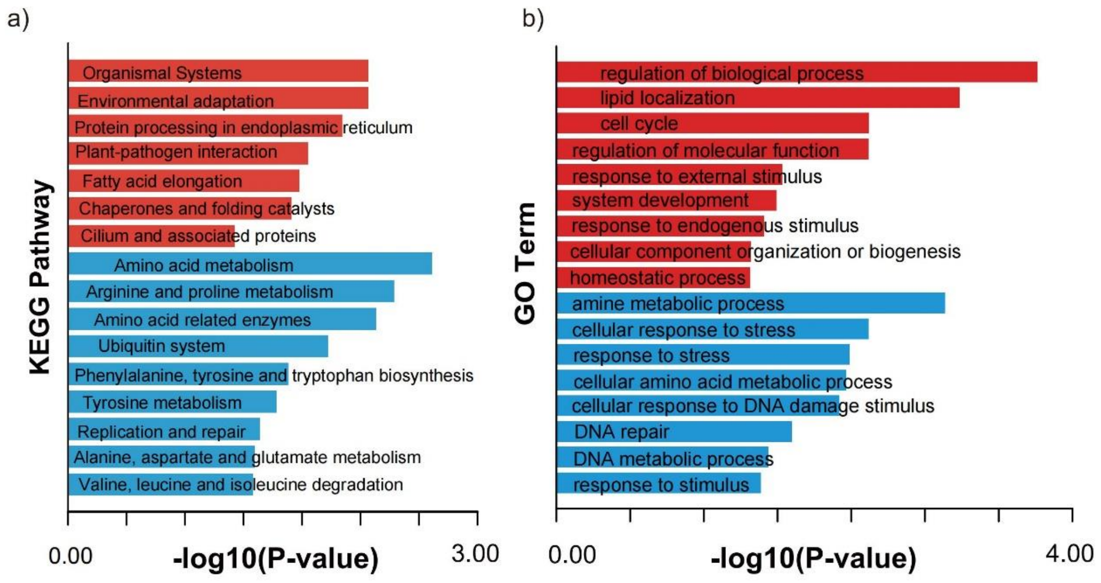

Supplementary Fig. 40. The KEGG (Kyoto Encyclopedia of Genes and Genomes) and Gene Ontology (GO) enrichment of the lost genes in each subgenome.

a) The KEGG enrichment of the lost genes in the A subgenome (red) and B subgenome (blue).

b) The Gene Ontology (GO) enrichment of the lost genes in the A subgenome (red) and B subgenome (blue).

A one-sided Fisher's exact test was adopted and adjustments were made for multiple comparisons with Benjamini and Hochberg method. Source data are provided as a Source Data file.

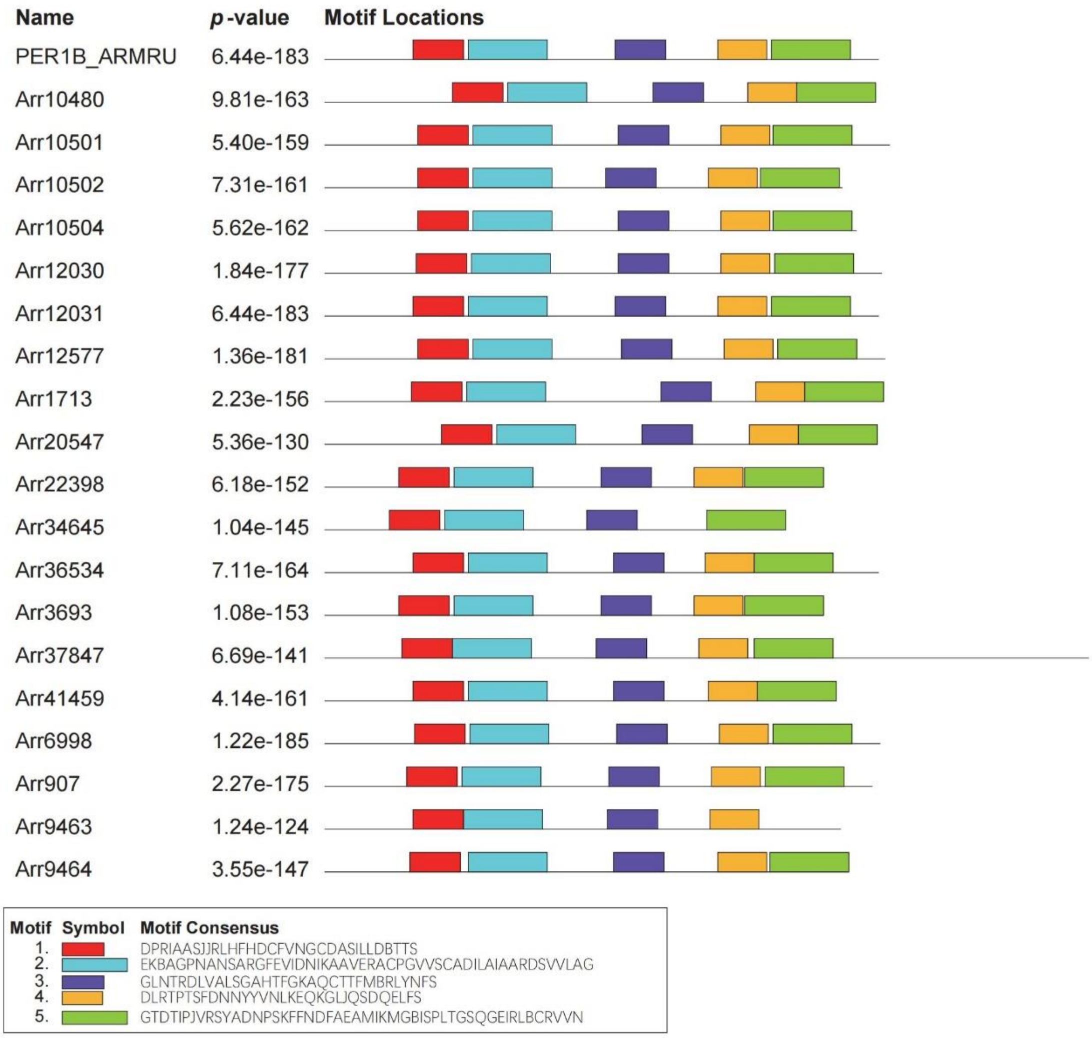

Supplementary Fig. 41. Motif analysis of the horseradish peroxidase proteins.

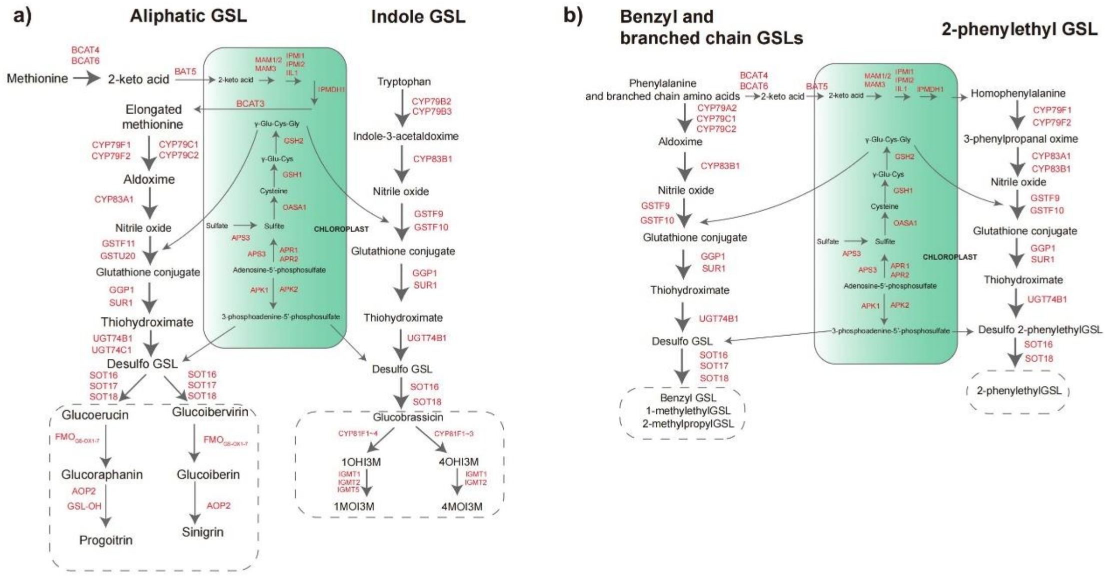

Supplementary Fig. 42. The biosynthesis of glucosinolates (GSLs) in *Arabidopsis thaliana* includes the methionine-derived pathway and the indole pathway (a), as well as the benzyl pathway, the branched-chain amino acid pathway, and the 2-phenylethyl pathway (b).

The enzymes and transporters are display in red font.

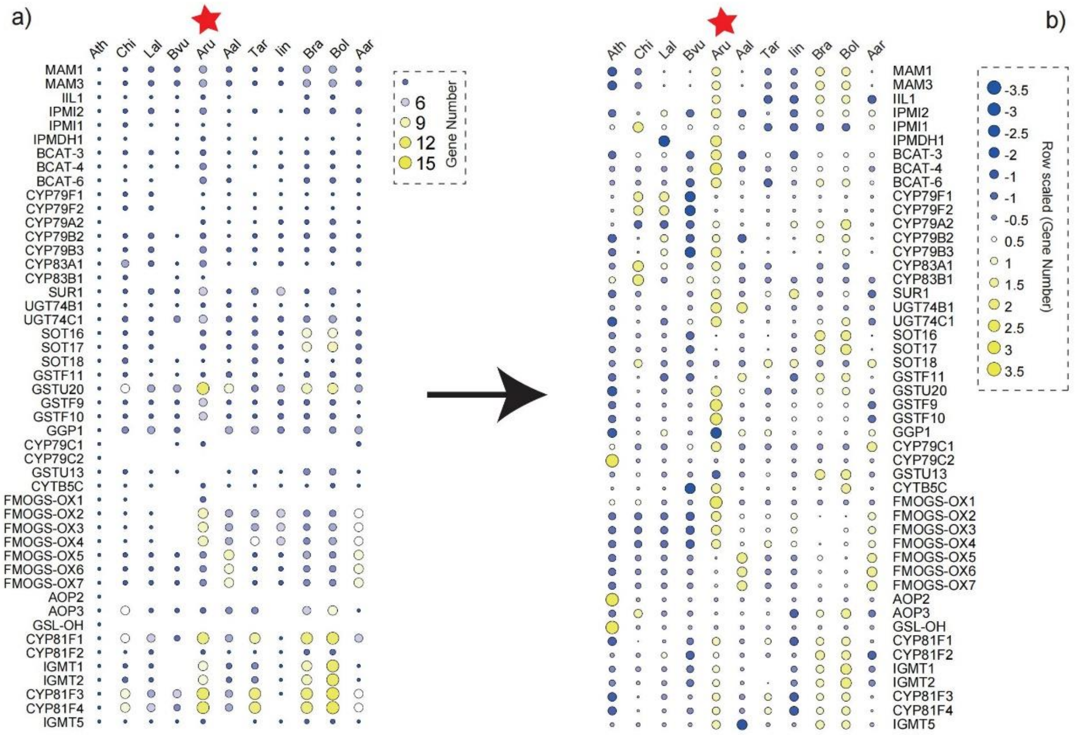

Supplementary Fig. 43. The numbers of genes in the glucosinolate biosynthesis in Brassicaceae species. a) The heatmap indicates the raw gene numbers. b) The heatmap indicates the row scaled gene numbers.

The 11 Brassicaceae species include *Arabidopsis thaliana* (Ath), *Barbarea vulgaris* (Bvu), *Cardamine hirsuta* (Chi), *Leavenworthia alabamica* (Lal), *Armoracia rusticana* (Aru), *Arabis alpina* (Aal), *Brassica rapa* (Bra), *Brassica oleracea* (Bol), *Isatis indigotica* (Iin), *Thlaspi arvense* (Tar) and *Aethionema arabicum* (Aar).

Source data are provided as a Source Data file.

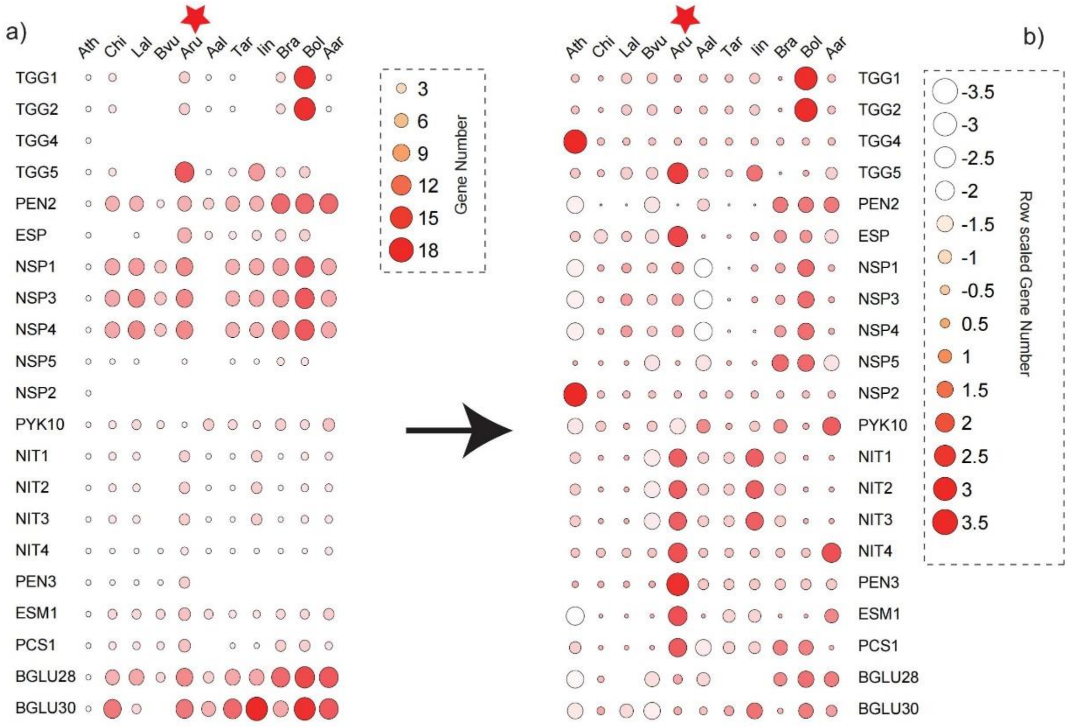

Supplementary Fig. 44. The numbers of genes in the glucosinolate breakdown in Brassicaceae species. a) The heatmap indicates the raw gene numbers. b) The heatmap indicates the row scaled gene numbers.

The 11 Brassicaceae species include *Arabidopsis thaliana* (Ath), *Barbarea vulgaris* (Bvu), *Cardamine hirsuta* (Chi), *Leavenworthia alabamica* (Lal), *Armoracia rusticana* (Aru), *Arabis alpina* (Aal), *Brassica rapa* (Bra), *Brassica oleracea* (Bol), *Isatis indigotica* (Iin), *Thlaspi arvense* (Tar) and *Aethionema arabicum* (Aar).

Source data are provided as a Source Data file.

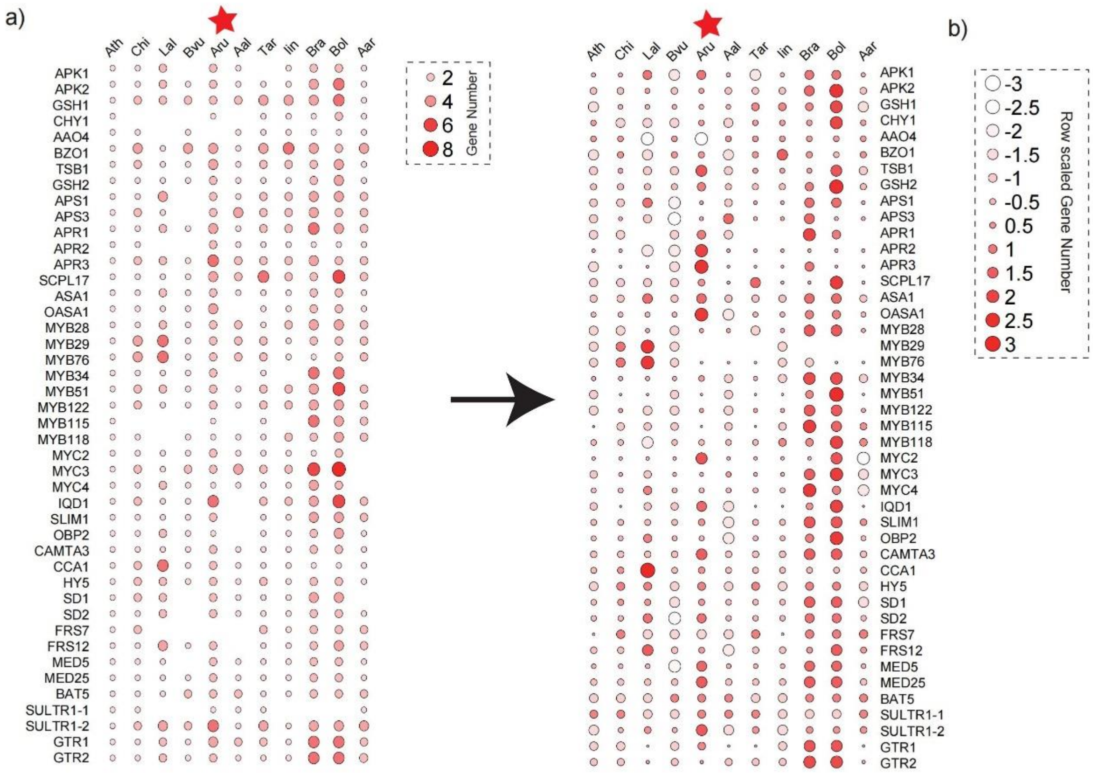

Supplementary Fig. 45. The numbers of genes involved in the regulation and transportation of glucosinolate biosynthesis in Brassicaceae species. a) The heatmap indicates the raw gene numbers. b) The heatmap indicates the row scaled gene numbers.

The 11 Brassicaceae species include *Arabidopsis thaliana* (Ath), *Barbarea vulgaris* (Bvu), *Cardamine hirsuta* (Chi), *Leavenworthia alabamica* (Lal), *Armoracia rusticana* (Aru), *Arabis alpina* (Aal), *Brassica rapa* (Bra), *Brassica oleracea* (Bol), *Isatis indigotica* (Iin), *Thlaspi arvense* (Tar) and *Aethionema arabicum* (Aar).

Source data are provided as a Source Data file.

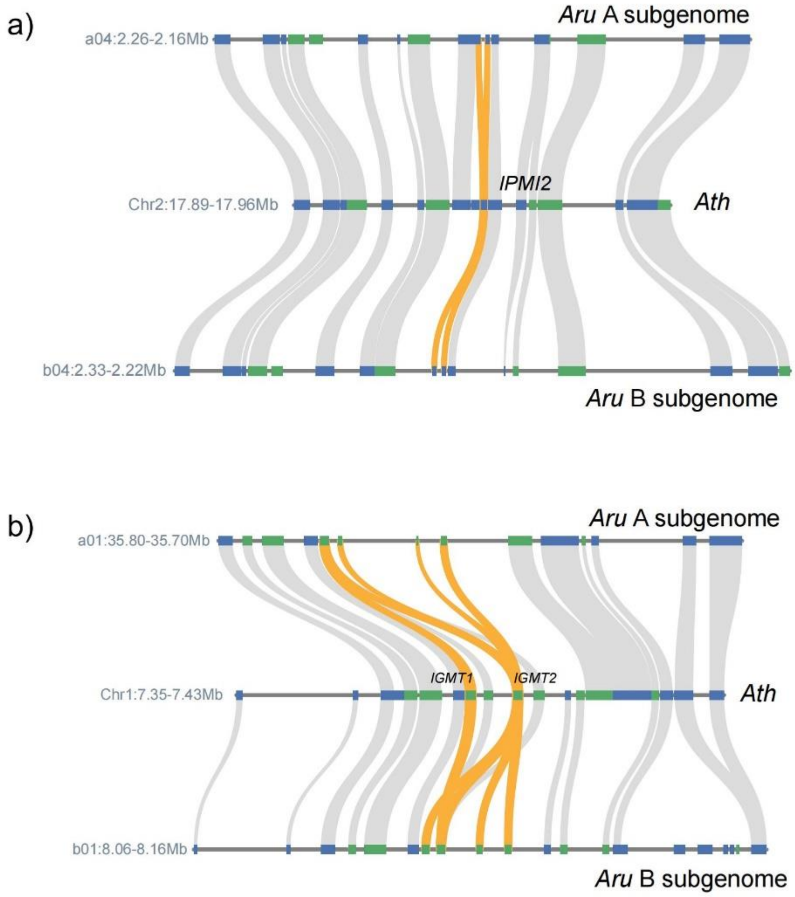

Supplementary Fig. 46. Microsynteny visualization of *IPMI2* (a) and *IGMT* genes (b) in *Armoracia rusticana* (Aru) and *Arabidopsis thaliana* (Ath).

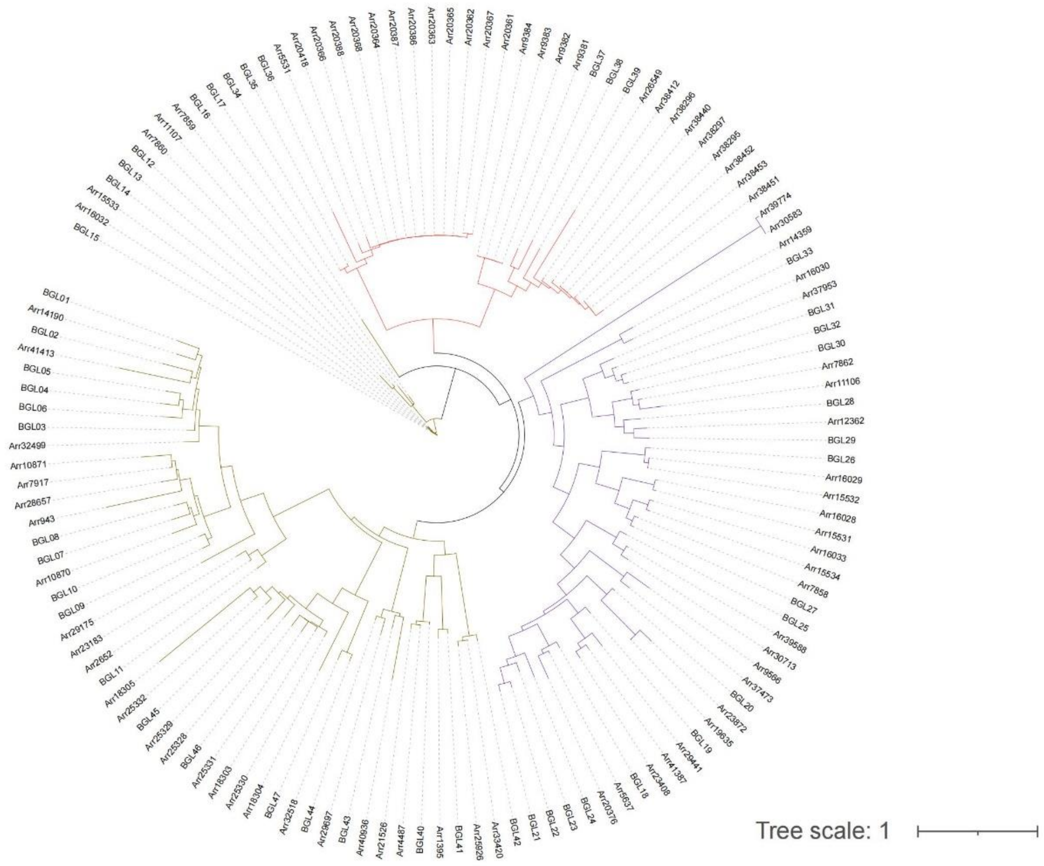

Supplementary Fig. 47. The phylogenetic tree of all the glucosyl hydrolase family 1 proteins from *Armoracia rusticana* and *Arabidopsis thaliana*.

The gene names starting with “Arr” were identified from the horseradish genome, and the others were from *A. thaliana*. blue clades: atypical myrosinase; red clades: typical myrosinase; brown clades: others.

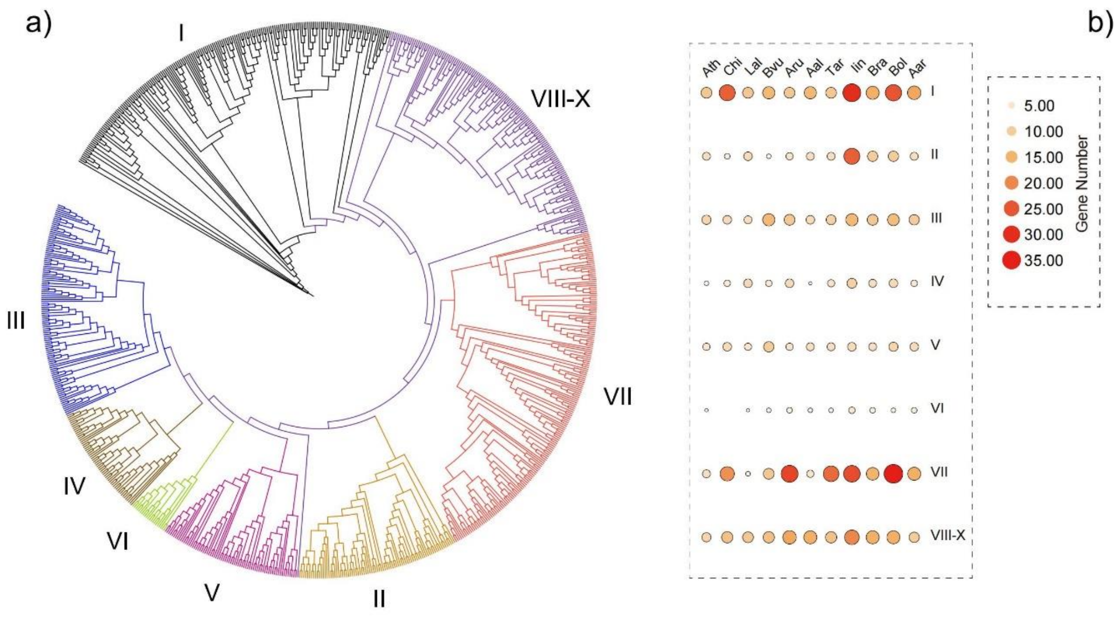

Supplementary Fig. 48. The analysis of glucosyl hydrolase family 1 (GH1) gene family in the 11 Brassicaceae species.

a) The phylogenetic tree of all the GH1 proteins from the 11 Brassicaceae species. The clades were divided based on the classification in *Arabidopsis thaliana*. b) The heatmap indicates the GH1 gene numbers in the 11 Brassicaceae species.

The 11 Brassicaceae species include *Arabidopsis thaliana* (Ath), *Barbarea vulgaris* (Bvu), *Cardamine hirsuta* (Chi), *Leavenworthia alabamica* (Lal), *Armoracia rusticana* (Aru), *Arabis alpina* (Aal), *Brassica rapa* (Bra), *Brassica oleracea* (Bol), *Isatis indigotica* (Iin), *Thlaspi arvense* (Tar) and *Aethionema arabicum* (Aar).

Source data are provided as a Source Data file

Supplementary Table 1. Statistics of all sequence data of *Armoracia rusticana*.

Library	Organ	Read No.	Base (G bp)	Coverage (×)	N50 (bp)	Average length (bp)
Nanopore	Leaf	2,409,515	52.28	82.20	29,011	20,471
Illumina	Leaf	373,293,962	55.99	88.03	-	150
Pacbio HiFi	Leaf	1,630,090	27.71	43.57	17,096	16,998
HiC	Leaf	2,251,039,938	337.66	530.91		150
Bisulfite sequencing	Leaf	320,032,058	48.00	75.47	-	150
Bisulfite sequencing	Stem	346,058,920	51.91	81.62	-	150
Bisulfite sequencing	Root	274,878,318	41.23	64.83	-	150
RNA-seq	Stem	41,021,114	6.15	-	-	150
RNA-seq	Root	40,805,650	6.12	-	-	150
RNA-seq	Leaf	40,469,630	6.07	-	-	150

Supplementary Table 2. Statistics of the initial assembly of the *Armoracia rusticana* genome using Nanopore reads.

**Metrics**	**Length (bp)**	**Count (#)**
N10	16162948	4
N20	14677515	8
N30	12419427	12
N40	9777353	18
N50	7947255	25
N60	5588164	34
N70	3841779	48
N80	2390741	69
N90	943257	110
Min.	37155	-
Max.	20541914	-
Ave.	2091952	-
Total	610850205	292

Supplementary Table 3. Statistics of the assembly of the *Armoracia rusticana* genome using Nanopore reads after scaffolding.

Metrics	Values (bp)
Total length	609255011
Longest contig	45489305
N10	43148698
N20	39467480
N30	37740391
N40	35945051
N50	35037176
N60	30677166
N70	29824229
N80	27492639
N90	17014146

Supplementary Table 4. Statistics of chromosomes of *Armoracia rusticana* assembled using Nanopore reads.

Chromosome	Scaffold No.	Contig No.	Size (bp)
a01	1	15	43837412
a02	1	30	43148698
a03	1	6	27509813
a04	1	15	35037176
a05	1	12	33743176
a06	1	7	34176234
a07	1	15	41619305
a08	1	10	37740391
b01	1	16	35624229
b02	1	27	39467480
b03	1	10	34471418
b04	1	9	38032616
b05	1	11	35252036
b06	1	8	27492639
b07	1	16	42987653
b08	1	5	27441495
Un-anchored	23	80	31673240
Total	39	292	609255011

Supplementary Table 5. Statistics of the *Armoracia rusticana* genome assembly based on Pacbio HiFi reads.

Metrics	Length (bp)
N10	39533315
N20	37746691
N30	34916798
N40	34620028
N50	33736477
N60	32737407
N70	29312911
N80	24350090
N90	13290677
Contig No.	108
Longest contig	44597498
Total	612393261

Supplementary Table 6. Statistics of the telomere-to-telomere genome assembly of *Armoracia rusticana.*

Chromosome	Contig No.	Gap No.	Telomere No.	Size (bp)
a01	1	0	2	44834628
a02	1	0	2	39142523
a03	1	0	2	33314349
a04	2	0	2	39616076
a05	1	0	2	30924649
a06	2	0	2	33326425
a07	1	0	2	37746691
a08	1	0	2	39533315
b01	3	0	2	34783792
b02	2	0	2	40748484
b03	2	0	1	36523187
b04	2	0	2	46531992
b05	1	0	2	34922554
b06	2	0	2	33226492
b07	1	0	2	33736477
b08	1	0	2	34620028
Un-anchored	84	na	na	16521048
Scaffold N50	na	na	na	36523187
Total	108	na	na	610052710

Supplementary Table 7. Statistics of telomere sequences in the T2T genome assembly of *Armoracia rusticana.*

Chromosome	Head			Tail		
	Start	End	Length (bp)	Start	End	Length (bp)
a01	1	1633	1633	44832271	44834628	2357
a02	1	2906	2906	39140160	39142523	2364
a03	1	3451	3451	33311304	33314349	3046
a04	1	2726	2726	39613163	39616076	2914
a05	1	3446	3446	30921466	30924649	3184
a06	1	2578	2578	33324036	33326425	2390
a07	1	2055	2055	37743387	37746691	3305
a08	1	3447	3447	39530919	39533315	2397
b01	1	2690	2690	34781761	34783792	2032
b02	1	2570	2570	40745709	40748484	2776
b03	na	na	na	36519481	36523187	3707
b04	1	2315	2315	46528381	46531992	3612
b05	1	2940	2940	34919379	34922554	3176
b06	1	1800	1800	33223500	33226492	2993
b07	1	2741	2741	33734058	33736477	2420
b08	1	3627	3627	34616629	34620028	3400

Supplementary Table 8. Statistics of candidate centromere regions in the *Armoracia rusticana* genome assembly.

Chromosome	Centromere regions		
	Start	End	Length (Mbp)
a01	14600000	17900000	3.3
a02	20300000	26000000	5.7
a03	12700000	16400000	3.7
a04	17100000	24600000	7.5
a05	10200000	14900000	4.7
a06	14000000	17700000	3.7
a07	13300000	16100000	2.8
a08	15000000	19400000	4.4
b01	20600000	23500000	2.9
b02	16600000	22900000	6.3
b03	20400000	23600000	3.2
b04	19300000	37800000	18.5
b05	20100000	23400000	3.3
b06	14800000	19100000	4.3
b07	19000000	22200000	3.2
b08	18900000	25600000	6.7

Supplementary Table 9. Quality assessment of the assembled genome of *Armoracia rusticana* using BUSCOs.

Metrics	Number	Percentage (%)
Complete BUSCOs (C)	1,605	99.44
Complete and single-copy BUSCOs (S)	520	32.22
Complete and duplicated BUSCOs (D)	1085	67.22
Fragmented BUSCOs (F)	3	0.19
Missing BUSCOs (M)	6	0.37
Total BUSCO groups searched	1,614	-

Supplementary Table 10. Quality assessment of the assembled genome of *Armoracia rusticana* based on aligning RNA-seq and DNA-seq reads.

Library type	Tissue type	Total read No.	Mapped reads No.	Mapping rate (%)
Illunima DNA	Leaf	372499180	370268435	99.40
Pacbio HiFi	Leaf	1630090	1628622	99.91
Nanopore (ONT)	Leaf	6398535	2397467	99.50
RNA-seq	Stem	66759587	64809674	97.08
RNA-seq	Root	66819262	64040652	95.84
RNA-seq	Leaf	64849187	63482807	97.89

Supplementary Table 11. Statistics of protein-coding genes in the *Armoracia rusticana* genome assembly.

Metrics	Gene number	Percentage (%)
Protein-coding	42,025	-
Interproscan	40,721	96.90
Uniprot (Swiss-prot)	32,911	78.31
NCBI (NR protein database)	41,145	97.91
Expressed	37,795	89.93
Annotated or expressed	41,855	99.60

Supplementary Table 12. Quality assessment of the gene set of the *Armoracia rusticana* genome using BUSCOs.

Metrics	Number	Percentage (%)
Complete BUSCOs (C)	1,587	98.33
Complete and single-copy BUSCOs (S)	568	35.19
Complete and duplicated BUSCOs (D)	1019	63.14
Fragmented BUSCOs (F)	5	0.31
Missing BUSCOs (M)	22	1.36
Total BUSCO groups searched	1,614	-

Supplementary Table 13. Protein-coding genes of 16 chromosomes in the *Armoracia rusticana* genome assembly.

Chromosome	Gene number
a01	3361
a02	2106
a03	2591
a04	2458
a05	2125
a06	2444
a07	2851
a08	2757
A genome sum	20693
b01	3378
b02	1997
b03	2937
b04	2469
b05	2310
b06	2406
b07	2794
b08	2822
B genome sum	21113
Others	219

Supplementary Table 14. Statistics of noncoding RNAs in the *Armoracia rusticana* genome.

Type	Copy No.
tRNAs	1143
microRNAs	557
snoRNAs	2600
rRNAs	7124
spliceosomal RNAs	195
Total	11619

Supplementary Table 15. Statistics of repetitive sequences of the *Armoracia rusticana* genome.

Type	Class	Count	Length of masked genome (bp)	%masked
LTR	*Copia*	52153	51420671	8.43
	Gypsy	165154	141580603	23.20
	unknown	100878	79114115	12.96
TIR	*CACTA*	24545	16355339	2.68
	Mutator	37367	17407897	2.85
	PIF_Harbinger	8069	3723241	0.61
	Tc1_Mariner	2418	1027239	0.17
	hAT	12288	6066858	0.99
non TIR	*Helitron*	112290	48731514	7.98
	Total interspersed	515162	365427477	59.88
Tandem Repeats	Overlapped with interspersed repeats	625525	91711741	15.03
	Not overlapped with interspersed repeats	261678	27832338	4.56
	Total	887203	119544079	19.59
Total repeats (interspersed plus tandem repeats)		1402365	393259815	64.44

Supplementary Table 16. Statistics of intact transposable elements in the *Armoracia rusticana* genome.

Type	Classification	Count
LTR	*Copia*	2,954
	Gypsy	5,252
	unknown	3,076
TIR	MITE	1,497
	DTA	919
	DTM	1,599
	DTC	845
noTIR	*Helitron*	835

Supplementary Table 17. Statistics of gene family analysis in 18 crucifer species.

Metrics	Number
Number of species	18
Number of genes	664387
Number of genes in orthogroups	618082
Number of unassigned genes	46305
Percentage of genes in orthogroups	93
Percentage of unassigned genes	7
Number of orthogroups	35190
Number of species-specific orthogroups	12029
Number of genes in species-specific orthogroups	68206
Percentage of genes in species-specific orthogroups	10.3
Mean orthogroup size	17.6
Median orthogroup size	8
G50 (assigned genes)	33
G50 (all genes)	31
O50 (assigned genes)	5016
O50 (all genes)	5740
Number of orthogroups with all species present	5409
Number of single-copy orthogroups	21

Notes: 18 species include species in Poaceae (*Oryza sativa*), Vitales (*Vitis vinifera*), Rosaceae (*Fragaria vesca*), Asterales (*Actinidia chinensis*, *Olea europaea*, *Coffea arabica*, *Solanum tuberosum*), and 11 Brassicaceae species (*Brassica rapa*, *B. oleracea*, *Isatis indigotica*, *Thlaspi arvense*, *Arabis alpina*, *Barbarea vulgaris*, *Armoracia rusticana*, *Leavenworthia alabamica, Cardamine hirsuta*, *Arabidopsis thaliana*, *Aethionema arabicum*).

Supplementary Table 18. Genome coverage statistics of A/B compartments in the *Armoracia rusticana* genome.

Iterm	A/B compartment	Total length (bp)	Percentage (%)
A-subgenome	A	118825000	39.84
A-subgenome	B	179450000	60.16
B-subgenome	A	123175000	41.73
B-subgenome	B	171975000	58.27
Whole-genome	A	242000000	40.78
Whole-genome	B	351425000	59.22

Supplementary references

1. Walker, B. J. *et al.* Pilon: An integrated tool for comprehensive microbial variant detection and genome assembly improvement. *PLoS One* **9**, e112963 (2014).
2. Campbell, M. S. *et al.* MAKER-P: A tool kit for the rapid creation, management, and quality control of plant genome annotations. *Plant Physiol.* **164**, 513–524 (2014).
3. Wang, Y. *et al.* MCScanX: A toolkit for detection and evolutionary analysis of gene synteny and collinearity. *Nucleic Acids Res.* **40**, e49 (2012).
4. Yang, Z. PAML 4: Phylogenetic analysis by maximum likelihood. *Mol. Biol. Evol.* **24**, 1586–1591 (2007).
5. Durand, N. C. *et al.* Juicer provides a one-click system for analyzing loop-resolution Hi-C experiments. *Cell Syst.* **3**, 95–98 (2016).
6. Jia, K. H. *et al.* SubPhaser: a robust allopolyploid subgenome phasing method based on subgenome-specific k-mers. *New Phytol.* **235**, 801–809 (2022).
7. Katoh, K., Misawa, K., Kuma, K. I. & Miyata, T. MAFFT: A novel method for rapid multiple sequence alignment based on fast Fourier transform. *Nucleic Acids Res.* **30**, 3059–3066 (2002).
8. Price, M. N., Dehal, P. S. & Arkin, A. P. FastTree 2 - Approximately maximum-likelihood trees for large alignments. *PLoS One* **5**, e9490 (2010).
9. Li, H. & Durbin, R. Fast and accurate short read alignment with Burrows-Wheeler transform. *Bioinformatics* **25**, 1754–1760 (2009).
10. Kim, D., Paggi, J. M., Park, C., Bennett, C. & Salzberg, S. L. Graph-based genome alignment and genotyping with HISAT2 and HISAT-genotype. *Nat. Biotechnol.* **37**, 907–915 (2019).
11. Li, H. Minimap2: Pairwise alignment for nucleotide sequences. *Bioinformatics* **34**, 3094–3100 (2018).
12. Simão, F. A., Waterhouse, R. M., Ioannidis, P., Kriventseva, E. V. & Zdobnov, E. M. BUSCO: Assessing genome assembly and annotation completeness with single-copy orthologs. *Bioinformatics* **31**, 3210–3212 (2015).
13. Ou, S., Chen, J. & Jiang, N. Assessing genome assembly quality using the LTR Assembly Index (LAI). *Nucleic Acids Res.* **46**, e126 (2018).
14. Schranz, M. E., Lysak, M. A. & Mitchell-Olds, T. The ABC’s of comparative genomics in the Brassicaceae: building blocks of crucifer genomes. *Trends in Plant Sci.* **11,** 535–542 (2006).
15. Guo, X. *et al.* Linked by ancestral bonds: Multiple whole-genome duplications and reticulate evolution in a brassicaceae tribe. *Mol. Biol. Evol.* **38**, 1695–1714 (2021)
16. Mandáková, T. *et al.* The more the merrier: Recent hybridization and polyploidy in Cardamine. *Plant Cell.* **25**, 3280–3295 (2013).
17. Lysak, M. A., Mandáková, T. & Schranz, M. E. Comparative paleogenomics of crucifers: Ancestral genomic blocks revisited. *Curr. Opin. Plant Biol.* **30**, 108–115 (2016).
18. Mandáková, T. & Lysak, M. A. Healthy roots and leaves: Comparative genome structure of horseradish and watercress. *Plant Physiol.* **179**, 66–73 (2019).
19. Sun, X. *et al.* Phased diploid genome assemblies and pan-genomes provide insights into the genetic history of apple domestication. *Nat. Genet.* **52**, 1423–1432 (2020).
20. Li, H. *et al.* Graph-based pan-genome reveals structural and sequence variations related to agronomic traits and domestication in cucumber. *Nat. Commun.* **13**, 682 (2022).
21. Jia, X. L. *et al.* miR156 switches on vegetative phase change under the regulation of redox signals in apple seedlings. *Sci. Rep.* **7**, 1–13 (2017).

22. Cheng, H., Concepcion, G. T., Feng, X., Zhang, H. & Li, H. Haplotype-resolved *de novo* assembly using phased assembly graphs with hifiasm. *Nat. Methods* **18**, 170–175 (2021).

23. Xu, M. *et al.* TGS-GapCloser: A fast and accurate gap closer for large genomes with low coverage of error-prone long reads. *Gigascience* **9**, giaa094 (2020).

24. Ou, S. & Jiang, N. LTR_retriever: A highly accurate and sensitive program for identification of LTR retrotransposons. *bioRxiv* 137141 (2017) doi:10.1101/137141.

25. Ellinghaus, D., Kurtz, S. & Willhoeft, U. LTRharvest, an efficient and flexible software for de novo detection of LTR retrotransposons. *BMC Bioinform.* **9**, 18 (2008).

26. Xu, Z. & Wang, H. LTR-FINDER: An efficient tool for the prediction of full-length LTR retrotransposons. *Nucleic Acids Res.* **35**, W265-W268 (2007).

27. Li, W. & Godzik, A. Cd-hit: A fast program for clustering and comparing large sets of protein or nucleotide sequences. *Bioinformatics* **22**, 1658–1659 (2006).

28. Altschul, S. F., Gish, W., Miller, W., Myers, E. W. & Lipman, D. J. Basic local alignment search tool. *J. Mol. Biol.* **215**, 403–410 (1990).

29. Bendall, M. L. *et al.* Telescope: Characterization of the retrotranscriptome by accurate estimation of transposable element expression. *PLoS Comput. Biol.* **15**, e1006453 (2019).

30. Zhang, Q. J. *et al.* The chromosome-level reference genome of tea tree unveils recent bursts of non-autonomous LTR retrotransposons in driving genome size evolution. *Mol Plant* **13**, 935–938 (2020).

31. Ma, J., Wing, R. A., Bennetzen, J. L. & Jackson, S. A. Plant centromere organization: a dynamic structure with conserved functions. *Trends Genet.* **23**, 134–139 (2007).

32. Keith Slotkin, R. The epigenetic control of the *Athila* family of retrotransposons in Arabidopsis. *Epigenetics* **5**, 483–490 (2010).

33. Naish, M. *et al.* The genetic and epigenetic landscape of the Arabidopsis centromeres. *Science* **374**, eabi7489 (2021).

34. Chen, Y. C., Liu, T., Yu, C. H., Chiang, T. Y. & Hwang, C. C. Effects of GC bias in next-generation-sequencing data on *de novo* genome assembly. *PLoS One* **8**, e62856 (2013).

35. Kozarewa, I. *et al.* Amplification-free Illumina sequencing-library preparation facilitates improved mapping and assembly of (G+C)-biased genomes. *Nat. Methods* **6**, 291-295 (2009).

36. Ou, S. *et al.* Benchmarking transposable element annotation methods for creation of a streamlined, comprehensive pipeline. *Genome Biol.* **20**, 275 (2019).

37. Benson, G. Tandem repeats finder: A program to analyze DNA sequences. *Nucleic Acids Res.* **27**, 573–580 (1999).

38. Han, X. *et al.* Two haplotype-resolved, gap-free genome assemblies for *Actinidia latifolia* and *Actinidia chinensis* shed light on the regulatory mechanisms of vitamin C and sucrose metabolism in kiwifruit. *Mol. Plant* **16**, 452–470 (2023).

39. Jayakodi, M. *et al.* The giant diploid faba genome unlocks variation in a global protein crop. *Nature* **615**, 652–659 (2023).

40. Vollger, M. R., Kerpedjiev, P., Phillippy, A. M. & Eichler, E. E. StainedGlass: Interactive visualization of massive tandem repeat structures with identity heatmaps. *Bioinformatics* **38**, 2049–2051 (2022).

41. Logsdon, G. A. *et al.* The structure, function and evolution of a complete human chromosome 8. *Nature* **593**, 101–107 (2021).

42. Walden, N., *et al*. Nested whole-genome duplications coincide with diversification and high

morphological disparity in Brassicaceae. *Nat Comm*. **11**, 3795 (2020).
43. Zhong, S., *et al*. Single-base resolution methylomes of tomato fruit development reveal epigenome modifications associated with ripening. *Nat Biotechnol* **31**, 154–159 (2013).
