# Supplementary material for: The allotetraploid horseradish genome provides insights into subgenome diversification and formation of critical traits
Source: Nat Commun. 2023 Jul 25;14:4102. doi: 10.1038/s41467-023-39800-y (PMC10368706; doi:10.1038/s41467-023-39800-y)
Supplement: Supplementary file 3 — Description of Additional Supplementary Files [file 41467_2023_39800_MOESM3_ESM.pdf]

**Description of Additional Supplementary Files**

File Name: Supplementary Data 1
Description: Statistics of genome coverage of identified transposable elements on 16 chromosomes of Armoracia rusticana.

File Name: Supplementary Data 2
Description: Statistics of different types of identified intact transposable elements on 16 chromosomes of Armoracia rusticana.

File Name: Supplementary Data 3
Description: Statistics of genome coverage and classification of long terminal repeat retrotransposons identified in the Armoracia rusticana genome.

File Name: Supplementary Data 4
Description: Statistics of the most abundant tandem repeat monomers in centromeric regions of the Armoracia rusticana genome.

File Name: Supplementary Data 5
Description: Coverage statistics of long terminal repeat retrotransposon families identified in centromeric regions of Armoracia rusticana.

File Name: Supplementary Data 6
Description: Gene Ontology (GO) analysis of rapidly evolving gene families at three different nodes of the Brassicaceae family tree.

File Name: Supplementary Data 7
Description: The statistics of the methylated cytosines in different tissues of Armoracia rusticana.

File Name: Supplementary Data 8
Description: The statistics of the differentially methylated genes in the Armoracia rusticana genome.

File Name: Supplementary Data 9
Description: Statistics of genes with homoeologous expression bias in the Armoracia rusticana genome.

File Name: Supplementary Data 10
Description: Statistics of genes encoding peroxidases in the Armoracia rusticana genome.

File Name: Supplementary Data 11
Description: The statistics of glucosinolate (GSL) related syntelogs of Arabidopsis thaliana in different crucifer species.

File Name: Supplementary Data 12
Description: The statistics of tandemly duplicated genes associated with GSL-related syntelogs of Arabidopsis thaliana in the Armoracia rusticana genome.

File Name: Supplementary Data 13

Description: Chromosomal position, gene expression and methylation levels of GSL related syntelogs in the Armoracia rusticana genome.

File Name: Supplementary Data 14
Description: The statistics of the glycoside hydrolase 1 (GH1) gene family in different crucifer species.

File Name: Supplementary Data 15
Description: Expression and methylation levels of the GH1 gene family in the Armoracia rusticana genome.

File Name: Supplementary Data 16
Description: Glucosinolate biosynthesis/metabolism-related genes with homologous expression bias in the Armoracia rusticana genome.

File Name: Supplementary Data 17
Description: Summary of the primer sequences used in this study.
