# Supplementary material for: The allotetraploid horseradish genome provides insights into subgenome diversification and formation of critical traits
Source: Nat Commun. 2023 Jul 25;14:4102. doi: 10.1038/s41467-023-39800-y (PMC10368706; doi:10.1038/s41467-023-39800-y)
Supplement: Supplementary file 21 — Reporting Summary [file 41467_2023_39800_MOESM21_ESM.pdf]

nature portfolio

Corresponding author(s): Fei Shen ; Martin A. Lysak

Last updated by author(s): Jun 24, 2023

Reporting Summary

Nature Portfolio wishes to improve the reproducibility of the work that we publish. This form provides structure for consistency and transparency in reporting. For further information on Nature Portfolio policies, see our Editorial Policies and the Editorial Policy Checklist.

Statistics

For all statistical analyses, confirm that the following items are present in the figure legend, table legend, main text, or Methods section.

n/a	Confirmed	
☐	☒	The exact sample size (*n*) for each experimental group/condition, given as a discrete number and unit of measurement
☐	☒	A statement on whether measurements were taken from distinct samples or whether the same sample was measured repeatedly
☐	☒	The statistical test(s) used AND whether they are one- or two-sided<br>*Only common tests should be described solely by name; describe more complex techniques in the Methods section.*
☐	☒	A description of all covariates tested
☐	☒	A description of any assumptions or corrections, such as tests of normality and adjustment for multiple comparisons
☐	☒	A full description of the statistical parameters including central tendency (e.g. means) or other basic estimates (e.g. regression coefficient) AND variation (e.g. standard deviation) or associated estimates of uncertainty (e.g. confidence intervals)
☐	☒	For null hypothesis testing, the test statistic (e.g. *F*, *t*, *r*) with confidence intervals, effect sizes, degrees of freedom and *P* value noted<br>*Give P values as exact values whenever suitable.*
☒	☐	For Bayesian analysis, information on the choice of priors and Markov chain Monte Carlo settings
☐	☒	For hierarchical and complex designs, identification of the appropriate level for tests and full reporting of outcomes
☒	☐	Estimates of effect sizes (e.g. Cohen's *d*, Pearson's *r*), indicating how they were calculated

Our web collection on statistics for biologists contains articles on many of the points above.

Software and code

Policy information about availability of computer code

Data collection

1. We conducted whole genome DNA sequencing using the Illumina, ONT and PacBio HiFi platforms. We constructed Illumina sequencing libraries (with an insert size of 300 bp) following the manufacturer's standard protocol and sequenced them on the Illumina HiSeq X platform. We constructed the ONT library according to the protocol provided with the genomic sequencing kit SQK-LSK109 (Oxford Nanopore Technologies, Oxford, UK). The long reads were generated on the PromethION platform, and high-accuracy base calling was conducted using Guppy (v4.0.15) software. For SMRT sequencing, the prepared SMRTbell libraries were sequenced on a PacBio Sequel II system.
2. The Hi-C library was then sequenced on the Illumina HiSeq X platform (Illumina) according to the PE150 strategy.
3. The paired-end RNA-seq libraries with insert sizes of 300 bp were constructed using the TruSeq Sample Preparation kit and sequenced on the Illumina HiSeq X platform.
4. We constructed bisulfite sequencing libraries for three replicates of different tissues, and conducted genome sequencing using the Illumina HiSeq X platform.

Data analysis

1. The software used was listed below: jellyfish (v2.3.1), GenomeScope 2.0, NextDenovo (v2.0), Pilon software (v1.24), MAKER-P pipeline (v2.29+), JCVI (v1.2.20), PAML (v4.9j), Juicer (v1.6), (3D-DNA) pipeline (v201008), HiFiasm (v0.17.7), TGS-gapcloser (v1.2.1), SubPhaser (v1.2), MAFFT (v7.490), FASTTREE (v2.1.11), BWA MEM (v0.7.17), Hisat2 (v2.2.1), Minimap2 (v2.24), BUSCO (v4.0) , tBlastn (v2.9.0+), AUGUSTUS (v3.5.0), SNAP (v1.0), Infernal (v1.1.3), Interproscan (v5.60-92.0), The Extensive de novo TE Annotator (EDTA) pipeline (v2.0.0), RepeatMasker (v4.1.2), LTR-retriever (v2.9.0), LTRharvest (v2.9.0), LTR_FINDER (v1.07), Orthofinder package (v2.4.0), MCMCTREE (v1.0), Raxml (v8.0.0), CAFE (v2.0) , Nanopolish (v0.8.4), deepTools (v3.5.1), Bismark (v0.24), HiCExplorer (v3.7.2), Fit-Hi-C (v2.05), MUSCLE (v5.1), StringTie (v2.1.5), MethPrimer program (v1.0).
2. All codes and pipelines for this study are openly available at Zenodo (https://doi.org/10.5281/zenodo.8058147).

For manuscripts utilizing custom algorithms or software that are central to the research but not yet described in published literature, software must be made available to editors and reviewers. We strongly encourage code deposition in a community repository (e.g. GitHub). See the Nature Portfolio guidelines for submitting code & software for further information.

Data

Policy information about availability of data

All manuscripts must include a data availability statement. This statement should provide the following information, where applicable:

- Accession codes, unique identifiers, or web links for publicly available datasets
- A description of any restrictions on data availability
- For clinical datasets or third party data, please ensure that the statement adheres to our policy

The genome sequence data and genome assembly generated in this study have been deposited in the National Genomics Data Center (https://ngdc.cncb.ac.cn/) under the accession code PRJCA009966. The genome assembly and annotation files and additional functional annotations are also available publicly at Figshare (https://doi.org/10.6084/m9.figshare.21780176.v1).

Human research participants

Policy information about studies involving human research participants and Sex and Gender in Research.

Reporting on sex and gender	N/A
Population characteristics	N/A
Recruitment	N/A
Ethics oversight	N/A

Note that full information on the approval of the study protocol must also be provided in the manuscript.

Field-specific reporting

Please select the one below that is the best fit for your research. If you are not sure, read the appropriate sections before making your selection.

☒ Life sciences ☐ Behavioural & social sciences ☐ Ecological, evolutionary & environmental sciences

For a reference copy of the document with all sections, see nature.com/documents/nr-reporting-summary-flat.pdf

Life sciences study design

All studies must disclose on these points even when the disclosure is negative.

Sample size	One sample's genome was sequenced. The young leaf, root, and stem tissues of horseradish was used for transcriptome sequencing in 2-4 biological replicates. For phylogenetic tree construction, numbers of the species was selected as described in the method section.
Data exclusions	1. For the genome assembly, we searched against bacterial database to rule out possible bacterial contamination. Besides, The organelle genome sequences was also ruled out from the contigs.<br><br>2. For the Illunima sequencing reads, we obtained the high-quality reads by removing the adapter sequences and filtering low-quality reads.
Replication	Replication was used for the RNA-seq, whole genome bisulfite sequencing and RT-qPCR experiment. Besides, various bootstrap replicates were used for the phylogenetic analysis.
Randomization	As we only conducted the genome assembly, the randomization was not required.
Blinding	No experimental validation works were carried out. Hence, blinding was not applicable.

Reporting for specific materials, systems and methods

We require information from authors about some types of materials, experimental systems and methods used in many studies. Here, indicate whether each material, system or method listed is relevant to your study. If you are not sure if a list item applies to your research, read the appropriate section before selecting a response.

Materials & experimental systems

n/a	Involved in the study
☒	☐ Antibodies
☒	☐ Eukaryotic cell lines
☒	☐ Palaeontology and archaeology
☒	☐ Animals and other organisms
☒	☐ Clinical data
☒	☐ Dual use research of concern

Methods

n/a	Involved in the study
☒	☐ ChIP-seq
☒	☐ Flow cytometry
☒	☐ MRI-based neuroimaging
